# Adenine Nucleotide Translocase: From Nucleotide Carrier to a Modulator of Mitochondrial Bioenergetics, Quality Control, and Cellular Communication

**DOI:** 10.3390/cells15070646

**Published:** 2026-04-02

**Authors:** Ursula Rauch-Kroehnert, Jacqueline Heger, Ulf Landmesser, Andrea Dörner

**Affiliations:** 1 Department of Cardiology, Angiology and Intensive Care Medicine, Deutsches Herzzentrum der Charité (DHZC) University Hospital Berlin, Campus Benjamin Franklin, 12203 Berlin, Germany; ursula.rauch@dhzc-charite.de (U.R.-K.);; 2 German Centre for Cardiovascular Research (DZHK), Partner Site Berlin, 10785 Berlin, Germany; 3 Institute of Physiology, Justus-Liebig-University, 35392 Giessen, Germany

**Keywords:** adenine nucleotide translocase, mitochondrial permeability transition pore, mitochondrial uncoupling, mitochondrial signaling, mitophagy, mitochondrial dynamics, mtDNA stability, dsRNA transport, immunometabolism, extracellular vesicles

## Abstract

**Highlights:**

**What are the main findings?**

- Adenine nucleotide translocase (ANT) acts as a mitochondrial multifunctional regulatory hub that stabilizes energetic balance, inner mitochondrial membrane integrity, and quality control beyond its canonical ADP/ATP exchange function.
- ANT integrates mitochondrial stress signals with cellular and systemic responses by regulating redox state, nucleic acid release, innate immune pathways, and intercellular communication through extracellular pathways.

**What is the implication of the main finding?**

- This framework positions ANT as a context-dependent regulator and organizer that stabilizes mitochondrial function under physiological conditions and coordinates adaptive signaling throughout the whole cell when homeostasis is challenged.
- Preserving or selectively modulating ANT-dependent regulatory interactions may support mitochondrial resilience, cytoprotection, and controlled stress communication.

**Abstract:**

Adenine nucleotide translocase (ANT) has traditionally been defined as the ADP/ATP exchanger of the inner mitochondrial membrane. However, accumulating mechanistic evidence reveals a substantially broader functional spectrum that extends beyond nucleotide transport. In this review, we integrate these advances into a unified conceptual framework that positions ANT isoforms as modulators of mitochondrial bioenergetics, quality control, and cellular communication. Beyond its canonical exchange activity, ANT influences permeability transition thresholds and membrane potential stability, participates in regulated uncoupling and redox control, and contributes to inner membrane organization and cristae integrity. ANT further modulates TIMM23-dependent protein import and PINK1–Parkin-mediated mitophagy, thereby shaping mitochondrial quality control decisions. In addition, ANT regulates mitochondrial nucleic acid release and inflammasome activation, linking bioenergetic imbalance to innate immune signaling. Emerging evidence for alternative subcellular localizations suggests that ANT-dependent signaling extends mitochondrial state information to extracellular and intercellular contexts. Collectively, these findings support an expanded view of ANT as a multifunctional modulator linking mitochondrial energetic state to stress adaptation, inflammatory signaling, and tissue-level communication.

## 1. Introduction

Mechanistically, adenine nucleotide translocase (ANT) is best known as the ADP/ATP exchanger of the inner mitochondrial membrane, providing the essential link between ATP synthesis in the matrix and ATP utilization in the cytosol [1]. Oxidative phosphorylation couples electron transfer through respiratory complexes I–IV to proton translocation across the inner mitochondrial membrane, generating a proton motive force that drives ATP synthase (complex V). ANT completes this system by catalyzing the electrogenic exchange of ADP^3−^ and ATP^4−^, thereby maintaining cytosolic ATP/ADP ratios and sustaining cellular work output. Structural and mechanistic studies established that ANT alternates between a cytosol-open “c-state” and a matrix-open “m-state”, enabling vectorial transport through sequential substrate binding and conformational switching [2]. Recent work on SLC25 family members supports a monomeric “ping-pong” exchange mechanism, reinforcing the view that tightly regulated substrate cycling underpins efficient nucleotide flux [3].

While ANT is essential for mitochondrial ATP export, its functional relevance extends well beyond nucleotide exchange. Recent reviews have provided important and insightful syntheses of selected aspects of ANT biology, particularly mitochondrial disease [4], post-translational regulation [5], and permeability transition [6]. Building on these contributions, the present review adopts a complementary integrative perspective that brings together bioenergetics, quality control, genome- and RNA-associated processes, intracellular signaling, and intercellular communication. In this framework, ANT is considered not only as a carrier, but also as a multifunctional regulator and organizer that connects mitochondrial energetic state with organelle adaptation, stress signaling, and immune-related responses. This concept emphasizes that ANT multifunctionality extends from transport and membrane regulation to signaling, structural organization, and communication across cellular compartments. A key principle underlying this broader functional spectrum is isoform specialization, as ANT isoforms differ in relative abundance, regulatory context, and developmental deployment.

## 2. ANT Isoforms: Expression, Regulation, and Functional Bias

**Figure 1:**
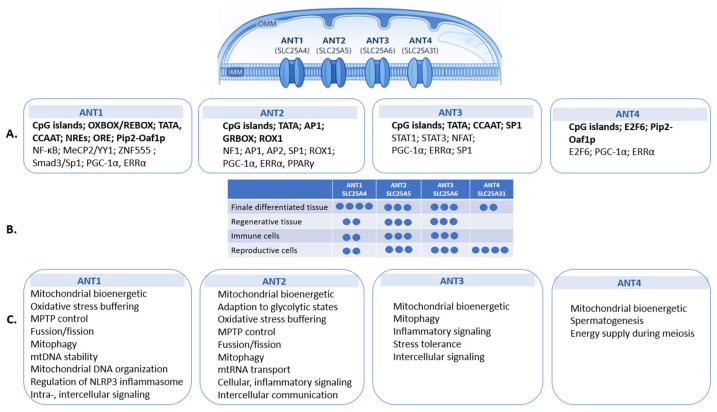
Overview of regulatory features, relative expression, and biological associations of mammalian ANT isoforms (ANT1–4). (**A**) Reported promoter-associated regulatory features, including CpG islands, transcription factor binding sites, and cis-regulatory elements, are shown for each isoform. (**B**) A semiquantitative scoring scheme illustrates relative expression levels across terminally differentiated, regenerative, immune, and reproductive cell contexts based on the data from ProteomicsDB. (**C**) Biological processes associated with each isoform are listed in separate summary boxes. These associations reflect the predominant functional contexts reported in the literature and highlight isoform-biased patterns rather than implying absolute functional exclusivity.

A key organizing principle underlying the diverse functions of ANT is isoform specialization. ANT is encoded by four nuclear genes that are frequently co-expressed within the same cell type, yet differ in relative abundance, tissue bias, and regulatory context (Figure 1) [7]. The four mammalian ANT isoforms share a conserved transport function but display only partial functional redundancy and are deployed in development- and tissue-biased patterns. Because rodents lack the ANT3 ortholog, isoform-specific findings from mouse and rat models should be interpreted with caution, as rodent ANT2 may partially subsume functions that in humans are distributed between ANT2 and ANT3 [8]. ANT1 and ANT2 both contain CpG-rich promoter regions but differ in their regulatory motifs and transcription factor inputs. ANT1 is linked to oxidative and differentiation-associated programs through OXBOX/REBOX elements and PGC-1α/ERRα signaling. By contrast, inflammatory repression via NF-κB and disease-associated regulation by MeCP2/YY1 or ZNF555 indicate context-sensitive downregulation under stress or pathology [9,10,11,12,13,14,15]. In contrast, ANT2 contains promoter elements associated with proliferative and glycolytic states, including AP1-, SP1-, and GRBOX-related regulation, consistent with its enrichment in metabolically flexible cells [9,16]. ANT3 appears to function as a more ubiquitously expressed housekeeping isoform, but it also responds to immune-related transcription factors such as STAT1, STAT3, and NFAT [17]. ANT4 is the most epigenetically restricted isoform, with promoter methylation silencing it in somatic tissues and hypomethylation permitting expression in the germline, where E2F6 contributes to developmental repression outside meiosis [18,19,20]. Together, these regulatory layers suggest that isoform-specific transcriptional and epigenetic control helps align ANT expression with distinct physiological and stress-responsive contexts.

Genetic studies in mice further support this functional specialization. ANT2 is critical during embryonic and perinatal development, whereas ANT1 becomes particularly important during postnatal tissue maturation and metabolic stress, especially in heart and skeletal muscle [21,22]. ANT3 shows broader distribution and immune-associated regulation [23]. In contrast, ANT4 fulfills a developmentally restricted role in the germline, where it is indispensable for meiotic progression and fertility [24]. Consistent with these genetic requirements, ANT isoform expression is dynamically regulated during development: ANT2 predominates in proliferative and undifferentiated cells, whereas ANT1 expression increases postnatally in tissues transitioning toward oxidative metabolism [25,26]. These observations establish ANT isoforms as stage-specific determinants of mitochondrial function rather than interchangeable nucleotide carriers.

This isoform logic provides an entry point for understanding how ANT links mitochondrial state to broader cellular behavior. By controlling nucleotide flux, ANT can tune membrane potential and redox state. In this way, it biases stress thresholds that influence mitochondrial permeability transition, mitophagy, and cell survival decisions [6,27]. In parallel, ANT contributes to processes that extend mitochondrial influence beyond energy supply, including mitochondrial genome organization, RNA handling, and signaling pathways that modulate proliferation, differentiation, and stress adaptation. Together, these findings support a conceptual shift in which ANT is viewed as a regulated integrator that both senses and propagates mitochondrial and cellular signals to coordinate intracellular and intercellular responses.

## 3. ANT in Mitochondrial Bioenergetics and Energetic Efficiency

### 3.1. Differential Contributions of ANT Isoforms to Mitochondrial Bioenergetics

ANT isoforms share a conserved nucleotide exchange mechanism but differ in transport kinetics, tissue distribution, and functional context. In cardiac rat and human mitochondria, ANT2 shows a higher maximal transport rate but lower ATP affinity than ANT1, consistent with deployment in high-flux conditions [28]. Functionally, ANT1 predominates in differentiated oxidative tissues, whereas ANT2 supports metabolically flexible and proliferative states. Genetic studies demonstrate partial compensation between isoforms, but also clear non-redundancy: ANT1 loss reduces mitochondrial ATP levels, whereas ANT2 deficiency produces a more pronounced ATP decline and glycolytic shift; combined deletion severely impairs mitochondrial ATP synthesis [29]. Thus, ANT isoforms define distinct energetic operating modes rather than interchangeable carrier capacity.

ANT2 also plays a key role under conditions of impaired oxidative phosphorylation. In stressed cardiomyocytes and in mitofusin-2 deficiency, ANT2 imports cytosolic glycolytic ATP into mitochondria, enabling reverse F1Fo-ATP synthase activity to maintain membrane potential (ΔΨm), a process constrained by Inhibitory factor (IF1) to prevent ATP depletion [30]. This reverse-mode operation highlights context-dependent directionality of ANT-mediated nucleotide flux.

Sub-mitochondrial distribution further supports functional specialization. While ANT1 and ANT2 are present in peripheral inner mitochondrial membrane regions, ANT2 is additionally enriched in cristae domains, where distinct regulatory interactions, including differential cyclophilin D association, have been reported [31].

Beyond nucleotide exchange, ANT contributes to respiratory chain organization. Respiratory complexes assemble into supercomplexes that enhance electron transfer efficiency and coupling [32,33]. In cardiac mitochondria, ANT inhibition destabilizes respirasome assemblies, showing the tight functional coupling between nucleotide exchange and respiratory chain architecture [34,35].

ANT abundance itself modulates mitochondrial efficiency in a non-linear manner. Higher carrier levels increase maximal nucleotide flux when transport is limiting but also raise basal proton conductance, reducing coupling efficiency, whereas lower ANT content is associated with tighter coupling at comparable respiratory capacity [36]. Taken together, isoform composition, localization, and carrier abundance set the operating range of mitochondrial nucleotide flux and coupling efficiency, thereby tailoring bioenergetic performance to developmental stage and metabolic demand.

### 3.2. ANT as a Mitochondrial Uncoupler and Regulator of Oxidative Stress

As mentioned above, ANT family members directly modulate mitochondrial coupling efficiency and oxidative stress through regulated proton conductance. Under conditions of metabolic or environmental stress—such as cold exposure, oxidative challenge, or hormonal stimulation—ANT can shift from nucleotide exchange to proton transport. This form of mild uncoupling partially dissipates the proton gradient, contributing to thermogenesis and limiting excessive mitochondrial reactive oxygen species (ROS) production. While transient uncoupling can be protective, sustained proton leak compromises ATP synthesis and promotes energetic failure.

In tissues lacking uncoupling protein 1 (UCP1), ANT1 mediates proton transport via a fatty acid–dependent cycling mechanism. Long-chain fatty acids and lipid peroxidation products such as 4-hydroxynonenal (4-HNE) activate this pathway, in which fatty acid anions cross the inner mitochondrial membrane and are reprotonated in the intermembrane space, dissipating ΔΨm [37,38,39]. ANT therefore functions as an inducible proton carrier whose activity depends on lipid environment and nucleotide availability.

ANT also complements the activity of UCP isoforms, particularly UCP2 and UCP3, which primarily modulate membrane potential, ROS production, and metabolic flexibility. When UCP2 or UCP3 activity is reduced, ANT-mediated proton leak increases, whereas combined inhibition of ANT and UCPs markedly reduces uncoupling [40,41,42,43]. In UCP3 knockout models, compensatory ANT upregulation helps preserve redox balance and supports fatty acid oxidation-linked adaptation.

By lowering electron pressure within the respiratory chain, mild uncoupling reduces ROS formation and supports maintenance of the NADH/NAD^+^ balance. However, excessive uncoupling decreases energetic efficiency and is particularly detrimental in tissues with high ATP demand, such as heart and brain. ANT-dependent uncoupling thus represents a context-dependent trade-off between redox protection and ATP yield.

ANT-dependent activity intersects functionally with UCP isoforms, particularly UCP3, whereas ANT2 has also been linked specifically to respiration-dependent interaction with UCP2. In cardiac ischaemia/reperfusion (I/R) models, H_2_O_2_ preconditioning induces UCP3 expression and confers cardioprotection characterized by preserved ΔΨm, reduced Ca^2+^ overload, and attenuated ROS production [44,45]. UCP3 has been reported to associate with ANT, and the protective effects of UCP3 overexpression are abolished by atractyloside, implicating ANT-dependent modulation of mPTP sensitivity. This coupling appears selective for UCP3 rather than UCP2. Notably, ANT1 overexpression in independent cardiac stress models also improves post-ischaemic recovery and limits mitochondrial injury, indicating that ANT can itself exert cardioprotective effects depending on expression level and context [46]. Together, these findings suggest that ANT integrates uncoupling activity and permeability control during cardiac stress, although the net impact of ANT modulation in I/R remains model-dependent.

ANT itself is both a target and mediator of oxidative stress. ROS and lipid-derived aldehydes, including 4-HNE, chemically modify ANT, increasing membrane permeability and impairing transporter function [47,48,49,50]. Conversely, ANT1 overexpression stabilizes ΔΨm and reduces aldehyde-associated injury in stress models [51]. During aging, ANT—particularly ANT1—shows increased oxidative carbonylation and lipid modification, correlating with declining mitochondrial efficiency and enhanced protonophoric activity [52,53].

These observations underscore that ANT is not only a nucleotide exchanger but a regulated contributor to mitochondrial proton leak and redox control, with protective or deleterious consequences depending on intensity and duration of activation.

## 4. ANT in Mitochondrial Integrity and Quality Control

Mitochondrial quality control is essential for preserving cellular homeostasis by maintaining bioenergetic capacity, redox balance, and selective removal of damaged organelles. Beyond its canonical role in nucleotide exchange, ANT has emerged as a central regulator of mitochondrial integrity that integrates energetic state with permeability control, protein turnover, and organelle surveillance.

### 4.1. ANT and the Mitochondrial Permeability Transition Pore

Current experimental evidence supports a model in which the mitochondrial permeability transition pore represents a stress-responsive inner membrane channel in which ANT acts as a key Ca^2+^- and stress-sensitive regulator and, in some contexts, a structural pore element, without being universally obligatory for permeability transition [54]. The mPTP is a dynamic inner membrane channel whose transient opening contributes to calcium and redox signaling, whereas sustained opening collapses mitochondrial membrane potential (ΔΨm), halts ATP synthesis, and initiates apoptotic or necrotic cell death. ANT-mediated control of mPTP interfaces with apoptotic signaling pathways. Consistent with this gatekeeper role, ANT1 overexpression stabilizes ΔΨm and limits pore opening in some stress models [51,55], whereas ANT1 dysregulation alters cell-death susceptibility and cytoskeletal organization in neuronal and cardiac systems, suggesting that impaired ANT-dependent permeability control contributes to degenerative vulnerability [56].

Historically, ANT was placed at the core of the pore together with CypD and outer-membrane VDAC. This model was supported by pharmacological studies, CsA-sensitive ANT–CypD interactions, and reconstitution experiments that identified a Ca^2+^-activated, CsA-inhibited channel [57,58,59]. ANT further operates within higher-order protein assemblies involving VDAC, hexokinase, mitochondrial creatine kinase, and the benzodiazepine receptor, which display permeability transition–like behavior and link metabolic flux to membrane permeability [57,60]. Residual pore activity has also been observed in the absence of ANT [61,62], implicating compensatory contributions from other carriers or ATP synthase. Together, these findings support a model in which ANT functions as a major regulator rather than an exclusive pore-forming unit. A comprehensive 2023 review concludes that the pore’s molecular identity remains unsettled [63]: strong evidence implicates both the F1FO ATP synthase (e.g., c-subunit or dimer interface) and ANT, with Cyclophilin D as a regulatory co-factor [64]. The prevailing view is that a small fraction of ATP synthase or ANT can convert into a non-selective channel under stress, but whether they form the same complex or distinct pores remains debated. New functional data suggest that ATP synthase is not invariably required as the pore-forming unit and may, in some contexts, exert inhibitory effects on mPTP opening [65].

Consequently, available data support the view that ANT can function as a dynamic gatekeeper of permeability transition rather than a universally obligate pore-forming subunit. By coupling nucleotide exchange to matrix Ca^2+^, redox state, and the lipid environment, it helps define the stress thresholds at which mitochondria may switch from adaptive, transient mPTP openings to catastrophic permeabilization and cell death. ANT-dependent control of permeability transition does not only influence cell-death susceptibility, but also alters membrane potential, redox balance, and inner membrane stability. These same parameters define the physicochemical conditions under which mitochondrial networks remain fusion-competent or shift toward fragmentation.

### 4.2. ANT at the Interface of Mitochondrial Fusion and Fission

**Figure 2:**
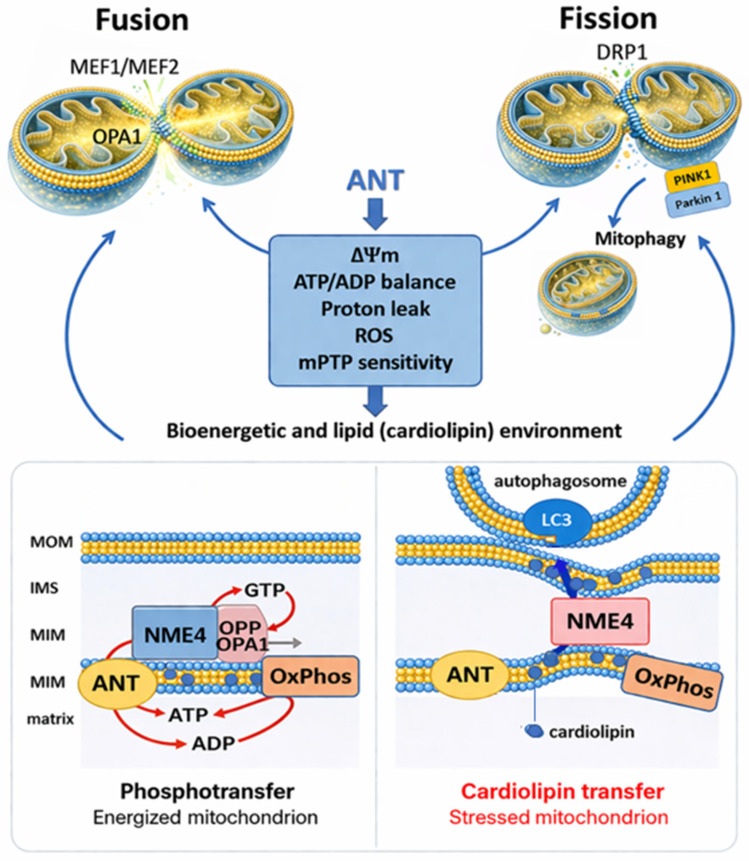
ANT integrates mitochondrial dynamics, bioenergetic control, and NME4-dependent lipid signaling upstream of mitophagy. The upper panel illustrates how ANT functionally positions mitochondrial bioenergetic state within the fusion–fission–mitophagy continuum. During mitochondrial fusion, MFN1/2 (outer membrane) and OPA1 (inner membrane) promote network connectivity and cristae organization. During fission, DRP1-mediated scission generates daughter mitochondria, enabling segregation of damaged units that can enter PINK1–Parkin–dependent mitophagy. ANT is a central inner membrane regulator that tunes ΔΨm, ATP/ADP balance, proton leak, ROS levels, and mPTP sensitivity, thereby shaping the bioenergetic and lipid (cardiolipin) environment that determines whether mitochondria remain fusion-competent or become mitophagy-prone. The lower panels depict the dual functional topology of the mitochondrial nucleoside diphosphate kinase NME4 at cardiolipin-rich inner membrane microdomains in proximity to ANT and OXPHOS. In healthy mitochondria (**left**), NME4 operates as an NDP kinase, using ANT-supplied ATP to regenerate GTP for OPA1, supporting inner membrane fusion and cristae dynamics (phosphotransfer mode). Under mitochondrial stress and mitophagy induction (**right**), NME4 switches topology and function, losing kinase activity and mediating cardiolipin transfer from the inner to the outer membrane. Externalized cardiolipin is recognized by the autophagic machinery, promoting selective mitophagic clearance of damaged mitochondria. Together, these mechanisms place ANT-centered bioenergetic control upstream of mitochondrial dynamics and lipid-based mitophagy signaling. Schematic illustration generated by the authors based on data discussed in the text and adapted from published work in [69].

Mitochondrial dynamics is governed by the balance between fusion and fission, which adapts network architecture to metabolic demand and quality control requirements. Outer membrane fusion is mediated by Mitofusin 1/2 (MFN1/2) and inner membrane fusion by Optic Atrophy 1 (OPA1), whereas Dynamin-Like Protein 1 (DRP1) drives fission and fragment segregation (Figure 2) [66,67]. ANT family members are not structural components of the fusion–fission machinery but regulate the underlying bioenergetic and physicochemical conditions that determine whether mitochondria fuse or shift toward fission.

Through control of ADP/ATP exchange, proton leak, and membrane potential (ΔΨm), ANT sets key upstream parameters for mitochondrial dynamics. Inner membrane fusion depends on OPA1 activity and an intact bioenergetic state [68]. ANT proteins operate in cardiolipin-rich inner membrane microdomains together with OPA1 and nucleoside diphosphate kinase D (NDPK-D/NME4), where ANT-supplied ATP supports local GTP generation for OPA1-dependent membrane remodeling and cristae organization (Figure 2) [69]. Loss of membrane potential disrupts this module, reduces fusion competence, and promotes fragmentation and mitophagy.

Conversely, increased ANT-dependent proton conductance and permeability transition sensitivity promote fission-prone states. Elevated proton leak, ROS production, and mPTP susceptibility shift network behavior toward fragmentation without requiring primary changes in core fission protein abundance [70]. Disruption of inner membrane regulatory assemblies that constrain ANT activity similarly enhances uncoupling and cell death sensitivity and secondarily promotes network fragmentation. Energetic stress states associated with MFN2 dysfunction show comparable features, including increased inner membrane leak and carrier dysregulation [69].

ANT abundance and isoform composition provide an additional scaling layer [70]. Changes in ANT protein levels track with respiratory capacity and mitochondrial mass and indirectly influence network structure through effects on energetic flux, redox balance, and cristae stability. Moderate ANT1 upregulation, for example, is sufficient to induce fragmentation and cristae distortion in muscle models, whereas ANT2 can help preserve ΔΨm under metabolic stress by importing glycolytic ATP, thereby stabilizing stressed mitochondrial populations.

Overall, ANT-controlled energetic and membrane parameters act as upstream constraints that bias mitochondrial networks toward fusion competence, adaptive fragmentation, or quality-control routing. By shaping membrane potential, inner membrane integrity, and fragmentation state, ANT therefore links mitochondrial network remodeling to the selective routing of dysfunctional organelles into mitophagy.

### 4.3. ANT as a Central Regulator of Mitophagy

Mitophagy eliminates dysfunctional mitochondria through autophagy-dependent turnover, thereby limiting the accumulation of bioenergetically compromised organelles associated with cancer, neurodegeneration, cardiovascular disease, and aging [71,72,73,74]. Genetic and mechanistic studies suggest that ANT proteins contribute critically to this process. Deletion of ANT1 or ANT2 markedly suppresses mitophagy despite mitochondrial depolarization, whereas pharmacological ANT inhibition promotes mitophagy, demonstrating a transport-independent regulatory role [75].

**Figure 3:**
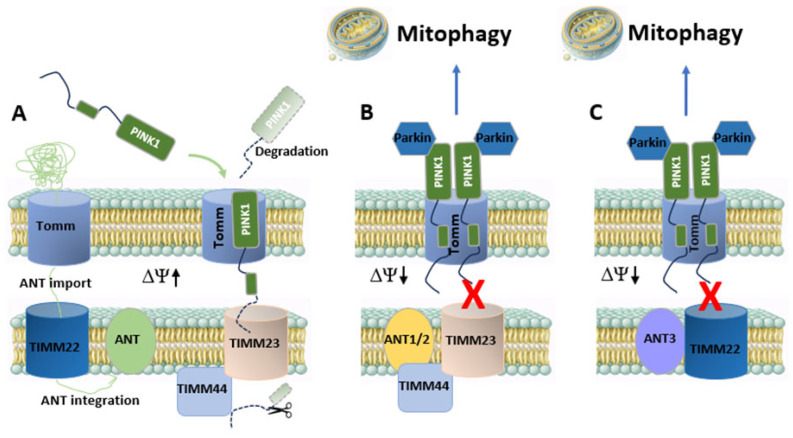
Model of ANT-Dependent Regulation of Mitochondrial Mitophagy. (**A**) Under basal conditions, ANT is inserted into the inner membrane through the TIMM22 pathway. PINK1 is imported via TOMM and TIMM23/TIMM44 translocases into polarized mitochondria (ΔΨm ↑), processed by inner membrane proteases, and degraded. (**B**) Under mitochondrial stress (ΔΨm ↓), ANT1/2 associates with TIMM23/TIMM44 and contributes to stress-induced inhibition of TIMM23-dependent import, resulting in PINK1 stabilization at the outer membrane, Parkin recruitment, ubiquitin signaling, and mitophagosome formation. (**C**) In proliferative or malignant contexts, ANT3 interacts with TOMM20/TIMM22 assemblies and limits PINK1 inner membrane import, likewise promoting accumulation of full-length PINK1 and enhanced Parkin-dependent mitophagy. Schematic illustration based on published literature cited in the text.

Mechanistically, ANT1/2 regulates mitophagy initiation through stress-responsive control of mitochondrial protein import. Upon mitochondrial depolarization, ANT1 and ANT2 are required for inhibition of the TIMM23 pre-sequence translocase, preventing continued matrix import and proteolytic processing of PINK1 and thereby stabilizing PINK1 at the outer mitochondrial membrane to enable Parkin recruitment (Figure 3) [75,76]. ANT1/2 physically associates with TIMM23 and its matrix-facing regulatory component TIMM44 in co-immunoprecipitation assays. Disease-associated ANT1 variants that fail to support mitophagy show reduced TIMM23/TIMM44 association. Loss of ANT1/2 markedly impairs PINK1 accumulation under depolarizing conditions, indicating that ANT acts upstream of canonical PINK1–Parkin signaling via TIMM44-associated translocase complexes [75]. Structural and regulatory functions rather than carrier activity are critical, as transport-deficient ANT1 variants restore mitophagy, whereas disease-associated mutants do not.

In addition, ANT1 abundance is controlled by ubiquitin-dependent quality control pathways that directly tune mitophagy efficiency. The deubiquitinase USP34 interacts with ANT1, limits its polyubiquitination, and stabilizes ANT1 protein levels. USP34 loss accelerates ANT1 degradation, reduces PINK1–Parkin–LC3 (receptor of the phagophore membrane) signaling, and impairs mitophagosome formation, whereas ANT1 overexpression restores mitophagy activity [77]. This regulatory axis places ANT1 functionally upstream of canonical mitophagy signaling and identifies ANT1 stabilization as a rate-limiting step in stress-induced mitochondrial quality control.

This pathway is further tuned by post-translational modification, as GSNOR-dependent denitrosylation of ANT1 at C160 is required to sustain cardiac mitophagy [78]. Environmental suppression of ANT1, such as after benzo[a]pyrene exposure, correspondingly reduces PINK1–Parkin signaling and mitophagic flux [79].

Isoform-linked pathways extend this framework. ANT2 is transcriptionally induced after nerve injury and promotes PINK1 stabilization and mitophagy, supporting mitochondrial clearance and axonal recovery [80]. In cancer, ANT3 associates with TOMM20/TIMM22-related assemblies, restricts PINK1 import, and enhances Parkin-dependent mitophagy independently of nucleotide transport, contributing to proteotoxic stress tolerance and proteasome inhibitor resistance [81]. Thus, distinct ANT isoforms can either lower or raise the threshold for mitophagy in a context-dependent manner, aligning organelle turnover with tissue-specific stress demands. All isoforms share conserved carrier architecture and cardiolipin binding. However, available data suggest that they may engage TIMM23/TIMM44 versus TOMM20/TIMM22 assemblies to different extents (Figure 3). ANT1/2 has been reported to associate with TIMM23/TIMM44-dependent import control in post-mitotic tissues, whereas ANT3 has been observed in proximity to TOMM20/TIMM22-containing complexes in proliferative contexts. However, strict isoform specificity has not yet been established. Current evidence is therefore most consistent with a shared ANT-dependent import-gating mechanism whose quantitative contribution may vary with isoform expression and cellular context.

ANT proteins are synthesized in the cytosol and inserted into the inner membrane via TOMM and TIMM22 complexes, placing them in close proximity to mitochondrial protein import machinery [82].

Recent work further shows that pathogenic ANT1 variants induce mitochondrial protein import stress and compensatory remodeling of TIMM22 pathway components, linking carrier import load, inner membrane proteostasis, and autophagy-related transcriptional programs. These findings support the concept that ANT is functionally embedded in mitochondrial import quality control, which can influence mitophagy thresholds under stress [83].

ANT-dependent mitophagy is closely linked to inner membrane integrity. MTFP1 stabilizes ANT-containing inner membrane assemblies and preserves cristae structure. Its loss destabilizes ANT complexes, increases membrane leakiness, sensitizes mitochondria to permeability transition, and reduces mitophagy competence independently of outer membrane fission pathways [70]. These findings link ANT-mediated import gating mechanisms with the structural stability of the inner membrane as a prerequisite for efficient mitophagy.

Together, these findings position ANT as an inner membrane damage sensor that gates PINK1–Parkin–dependent mitophagy through protein import control rather than nucleotide exchange, thereby coupling mitochondrial energetic state to selective organelle turnover and network remodeling.

## 5. ANT at the Interface of the Mitochondrial Genome and RNA

Through structural, metabolic, and regulatory interactions at the inner mitochondrial membrane, ANT links bioenergetic state to the stability and organization of mitochondrial DNA (mtDNA) and associated transcriptional machinery.

### 5.1. ANT Mutations and mtDNA Instability

Several heterozygous missense mutations in the ANT1 gene have been identified in patients with autosomal dominant progressive external ophthalmoplegia, mitochondrial myopathy, and cardiomyopathy [84,85,86,87]. Among these, substitution of a conserved alanine by proline at position 114 (A114P) is associated with secondary accumulation of mtDNA deletions in postmitotic tissues [85]. Although the precise mechanism underlying this phenotype remains unresolved, impaired ANT1 function has been proposed to disturb mitochondrial adenine nucleotide homeostasis. This may indirectly impair mtDNA replication fidelity. Beyond nucleotide imbalance, multiple secondary mechanisms may exacerbate ANT1-associated mtDNA instability. These include increased susceptibility to oxidative damage due to protein misfolding and elevated oxidative stress, all of which negatively affect mtDNA maintenance. Consistent with this model, analogous mutations in the yeast ANT homolog AAC2, including A128P, A106D, and M114P, destabilize mtDNA by uncoupling the inner mitochondrial membrane, impairing electron transport, reducing membrane potential (ΔΨm), and increasing sensitivity to uncoupling agents [87].

Animal models further support a protective role for ANT1 in mtDNA maintenance. ANT1-deficient mice display elevated levels of mtDNA deletions, particularly in tissues with limited antioxidant capacity [88]. In contrast, skeletal muscle with robust antioxidant defenses exhibits fewer mtDNA rearrangements, underscoring the contribution of oxidative stress to mtDNA instability and highlighting ANT1-dependent preservation of mitochondrial genome integrity under metabolic stress.

Collectively, ANT1 mutations promote mtDNA instability by perturbing inner membrane integrity and redox balance, thereby predisposing postmitotic tissues to mtDNA damage and deletions. Beyond preserving mtDNA stability, ANT has also been implicated in the organization and maintenance of mitochondrial nucleoids.

### 5.2. ANT Coordinates Mitochondrial Nucleoid Organization and Stress-Responsive Gene Regulation

**Figure 4:**
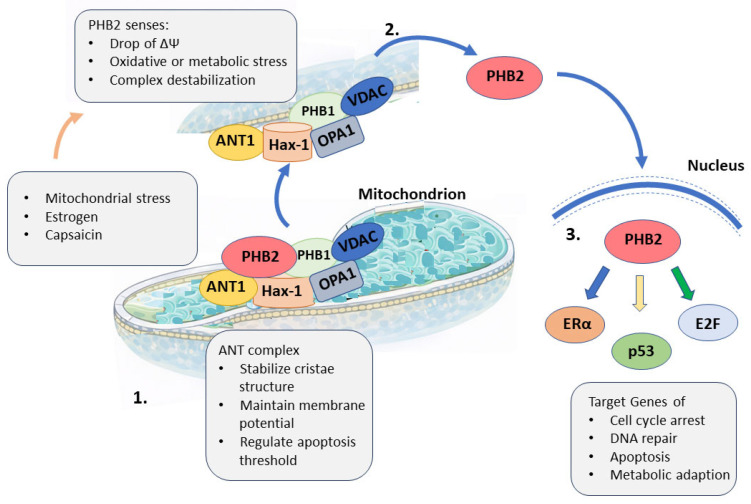
Proposed prohibitin-2 (PHB2)–mediated mitochondrial-to-nuclear signaling. PHB2 forms a multiprotein complex with PHB1, ANT, VDAC, Hax-1, and OPA1 at the inner mitochondrial membrane, where it stabilizes cristae structure, maintains membrane potential, and regulates the apoptotic threshold (1). Upon mitochondrial stress, including membrane depolarization, oxidative or metabolic stress, or destabilization of the complex, PHB2 dissociates from the membrane complex (2) and translocates to the nucleus (3). In the nucleus, PHB2 acts as a transcriptional coregulator by interacting with ERα, p53, and RB/E2F complexes, thereby modulating the expression of genes involved in cell cycle arrest, DNA repair, apoptosis, and metabolic adaptation. Schematic illustration based on published literature cited in the text.

Evidence increasingly links ANT to mitochondrial genome organization through its presence in nucleoid-associated inner membrane domains and its interaction with structural scaffold proteins. Mitochondrial nucleoids are membrane-anchored nucleoprotein complexes that coordinate mtDNA replication and transcription and are functionally coupled to inner membrane activity. Core nucleoid components include TFAM, POLRMT, POLG, Twinkle, and mtSSB [89]. Proteomic and fractionation studies place ANT isoforms, including ANT1, together with prohibitin (PHB) complexes in nucleoid-associated membrane regions, where they are proposed to contribute to membrane anchoring and local nucleotide supply [90]. Beyond structural proximity, ANT participates in stress-responsive mitochondrial–nuclear signaling through dynamic inner membrane scaffold complexes. ANT associates with PHB2, PHB1, VDAC, hematopoietic cell-specific Lyn substrate 1-associated protein X1 (Hax-1), and OPA1 in multiprotein assemblies that stabilize cristae structure, maintain ΔΨm, and modulate apoptotic sensitivity (Figure 4) [91,92]. Disruption of these complexes during mitochondrial stress promotes PHB2 release from the inner membrane. Once relocalized, PHB2 translocates to the nucleus, where it acts as a transcriptional coregulator through interactions with p53, estrogen receptor α, and RB/E2F complexes to modulate gene programs involved in cell cycle control, apoptosis, and metabolic adaptation [93]. In parallel, prohibitin assemblies also interface with mitophagy and mitochondrial protease systems, linking ANT-associated membrane domains to quality control pathways [94,95]. These observations connect ANT-containing inner membrane assemblies with nucleoid organization, stress-induced nuclear transcriptional responses, and broader signaling outputs. ANT is therefore positioned to link mitochondrial structural state and genome-associated functions with adaptive gene regulation.

## 6. ANT as a Signaling and Immunometabolic Hub

Mitochondrial genome organization and RNA handling encode mitochondrial state at the molecular level, but their functional relevance depends on how these signals are converted into cellular signaling networks. By controlling nucleotide flux, membrane potential, and permeability, ANT links mitochondrial bioenergetics to stress signaling, immune function, and immunometabolic adaptation. The following section considers these ANT-dependent pathways across three interconnected layers: kinase-responsive regulation, nucleic acid- and inflammasome-associated danger signaling, and downstream immunometabolic adaptation.

### 6.1. ANT as an Integrator of Intracellular Signaling and Stress Responses

Multiple signaling pathways converge on ANT, positioning it as a mitochondrial integration node where kinase, metabolic, and calcium-dependent signals are translated into changes in nucleotide exchange, membrane potential (ΔΨm), and permeability. ANT-dependent mitochondrial state changes also feed back into cytosolic signaling networks. ANT thus operates within a bidirectional signaling axis as both a downstream target and an upstream modulator of stress and survival pathways. The signaling mechanisms discussed below are organized into three interconnected axes: kinase-dependent regulation, post-translational control of ANT activity, and ANT-dependent feedback to calcium- and survival-associated signaling pathways.

Among these pathways, the PI3K/AKT axis is the most mechanistically defined ANT-linked signaling route. Under stress conditions such as ischemia, AKT translocates to mitochondria and associates with ANT [96]. Downstream, AKT regulates GSK-3β, which phosphorylates ANT—most prominently at serine 62—thereby modulating nucleotide transport, ΔΨm stability, oxidative stress, and mPTP sensitivity [97,98]. In this direction, kinase signaling tunes ANT-dependent mitochondrial function. In the opposite direction, ANT-controlled ΔΨm and ROS output influence AKT/GSK-3β pathway activity and permeability transition thresholds, establishing functional feedback between mitochondrial carrier state and survival kinase signaling [55]. In addition, ANT2 knockdown disrupts the HER2–HSP90 complex, accelerates receptor degradation, and suppresses PI3K/AKT signaling [99]. This bidirectional coupling is particularly evident in cardiomyocytes with elevated ANT1 expression. ANT1 overexpression enhances AKT phosphorylation and establishes a cytoprotective feedback circuit under hypoxic stress [100]. A key amplifier is HSP27. Increased HSP27 release activates TLR4 signaling and further augments AKT activity, reinforcing ΔΨm preservation, reducing ROS and caspase activation, and promoting HIF-1α–dependent VEGF and ANT1 expression [55,100]. Thus, ANT1-dependent mitochondrial adaptation actively reinforces pro-survival signaling rather than merely responding to upstream kinase input. Additional kinase pathways—including SRC family kinases and receptor tyrosine kinase-dependent networks—also regulate ANT through phosphorylation or complex stabilization, while ANT-dependent bioenergetic and redox outputs feed back into proliferation and migration signaling modules [55,101,102,103]. Because isoform expression and signaling context vary across tissues, these interactions should be interpreted as context-dependent rather than strictly isoform-fixed.

Post-translational modification provides an additional regulatory layer within this two-way signaling architecture. In prostate cancer cells, the PAK6–SIRT4–ANT2 axis fine-tunes ANT2 activity and mitochondrial apoptosis [104]. PAK6 promotes ubiquitin-dependent SIRT4 degradation and directly modifies ANT2 by phosphorylation (Thr107) and increased acetylation (Lys105), resulting in apoptosis suppression and tumor-supportive signaling. A broader overview of ANT post-translational modifications is provided in a recent review [5].

ANT-linked signaling is further integrated with calcium handling and excitation–contraction coupling. Altered sarcoplasmic reticulum Ca^2+^ buffering modifies mitochondrial gene expression, including ANT transcripts [105]. Lipid stress increases ANT-dependent ROS production and promotes oxidative RyR2 modification and sarcoplasmic reticulum Ca^2+^ leak [106], whereas pharmacological ANT inhibition suppresses sarcoplasmic reticulum Ca^2+^ release in skeletal muscle [107]. Consistent with this coupling, cardiac ANT1 overexpression is associated with enhanced contractile performance and improved calcium cycling, including increased SERCA2a expression and sarcoplasmic reticulum Ca^2+^ uptake capacity [108,109,110].

Collectively, this evidence supports a two-directional signaling architecture in which ANT is both shaped by kinase and stress pathways and, in turn, reshapes downstream survival and calcium-dependent signaling outputs.

### 6.2. ANT and microRNA-Dependent Regulation

ANT is also embedded in post-transcriptional regulatory networks formed by microRNAs that fine-tune isoform expression and function (Table 1). miR-2861 directly binds the coding sequence of ANT1, suppressing its expression and sensitizing cardiomyocytes to necrotic cell death under oxidative stress [111]. Conversely, inhibition of miR-2861 protects against H_2_O_2_-induced necrosis and ischemia–reperfusion injury in vivo.

ANT2 participates in distinct miRNA-dependent circuits. Proteomic analyses identified ANT2 as a binding partner of miR-29b, which co-localizes with ANT2 in perinuclear clusters and influences nuclear architecture during mitosis [112]. Loss of ANT2 phenocopies miR-29b inhibition, leading to abnormal nuclear morphology and impaired nuclear uptake of endogenous miR-29b, revealing a role for ANT2 in coordinating mitochondrial function with cell-cycle–dependent nuclear organization. These findings suggest that ANT2 may contribute not only to intramitochondrial nucleoid-associated processes (see Section 5) but also to miRNA-dependent coordination between mitochondrial state and nuclear architecture.

In cancer, ANT2–miRNA interactions critically shape oncogenic signaling. ANT2 suppression induces miR-636, a tumor-suppressive miRNA targeting Ras, thereby inhibiting proliferation and tumor growth in hepatocellular carcinoma models [113]. In parallel, ANT2 knockdown reduces oncogenic miRNAs including miR-19a, miR-96, miR-21, and miR-221/222, restoring SOCS1 expression, suppressing STAT3 activity, and attenuating PI3K/AKT signaling [114,115,116]. ANT2 upregulation in sorafenib-resistant HCC cells promotes cancer-initiating cell traits and metastasis, whereas restoration of miR-137 reverses these phenotypes [117]. These data position ANT2 as a node at which miRNA networks intersect with metabolic reprogramming, survival, and therapy resistance.

Pathogens also exploit this axis: during human cytomegalovirus infection, the viral miRNA hcmv-miR-UL36-5p suppresses ANT3 expression, thereby inhibiting apoptosis and promoting viral persistence [118]. Together, these data support a reciprocal relationship between ANT isoforms and miRNA networks, in which miRNAs modulate ANT expression and activity, while ANT-dependent mitochondrial states reshape miRNA-controlled programs linking metabolism, nuclear signaling, oncogenesis, and host–pathogen responses. These post-transcriptional circuits converge functionally with inflammatory cytokine pathways, which regulate ANT expression and mitochondrial stress responses at the transcriptional and signaling level.

### 6.3. Cytokine-Dependent Regulation and Inflammasome Control

Cytokine signaling is a major interface through which inflammatory cues are translated into mitochondrial functional changes. ANT isoforms represent key mitochondrial targets within this axis, converting cytokine-driven transcriptional and post-translational signals into changes in bioenergetics, redox balance, and permeability control.

Pro-inflammatory cytokines—including TNFα, IL-6, IL-1β, and interferons—consistently suppress ANT1 expression, largely via NF-κB–dependent transcriptional repression (Table 1) [11,100]. Functionally, this is associated with reduced ATP production, ΔΨm destabilization, increased ROS formation, and enhanced susceptibility to necrotic cell death. In this setting, inflammatory signaling acts upstream of ANT1 to lower mitochondrial stress tolerance thresholds.

Anti-inflammatory and immune-modulatory cytokines can exert opposing effects. IL-4 induces ANT1 in cardiomyocytes and ANT3 in T cells through kinase- and NF-κB-linked pathways and promotes cell survival programs [119]. IFN-γ regulates ANT3 via STAT1 signaling, with IL-4/STAT6 activity acting antagonistically [23,119]. These patterns indicate context-dependent, isoform-biased cytokine control rather than uniform regulation across ANT family members.

TGF-β signaling adds a further isoform-divergent layer. In senescent cells, TGF-β represses ANT2 transcription via NF1/Smad4 promoter complexes, increasing ROS and apoptosis susceptibility [120]. In contrast, elevated ANT1 levels in cardiomyocytes are associated with preserved ΔΨm and reduced TGF-β-linked apoptotic signaling, together with altered downstream SMAD responses [121]. These findings support differential integration of TGF-β signaling across ANT isoforms.

**Figure 5:**
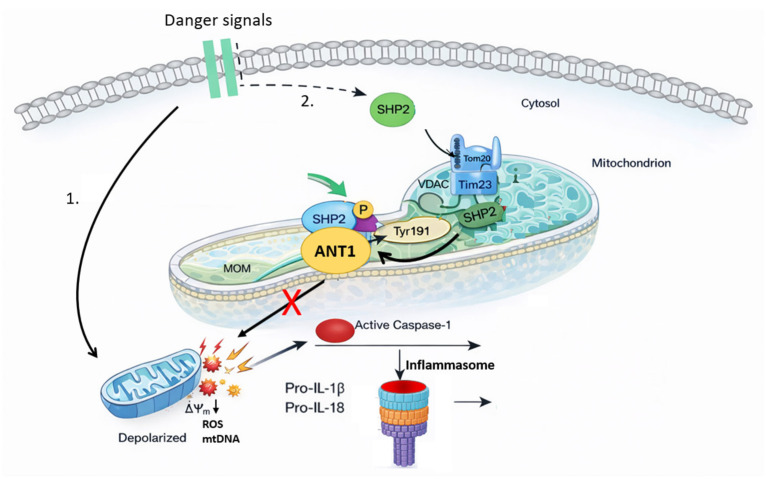
Proposed Model of SHP2–ANT1 Interaction in the Regulation of Mitochondrial ROS and Inflammasome Signaling. 1. Danger signals trigger mitochondrial depolarization, ROS production, and mitochondrial DNA release, thereby promoting NLRP3 inflammasome assembly and interleukin cleavage. 2. In parallel, these stimuli induce recruitment of the tyrosine phosphatase SHP2 to mitochondria through the TOMM20/TIMM23 import machinery. At the inner mitochondrial membrane, SHP2 associates with and dephosphorylates ANT1 (Tyr191), stabilizing mitochondrial membrane potential and limiting further ROS and mtDNA release. This ANT1–SHP2 axis establishes a negative feedback loop that restrains caspase-1 activation and dampens IL-1β and IL-18 maturation, thereby preventing excessive inflammasome-driven inflammation. Schematic illustration generated by the authors and adapted from published data in [122].

ANT also acts in the reverse direction by controlling mitochondrial danger signaling toward innate immune pathways. Reduced ANT1 function or destabilized ANT-dependent membrane control promotes release of mitochondrial danger-associated molecular patterns that activate the NLRP3 inflammasome. The tyrosine phosphatase SHP2 provides a counter-regulatory feedback mechanism: upon inflammasome activation, SHP2 translocates to mitochondria, associates with ANT1, and dephosphorylates it, stabilizing ΔΨm and limiting ROS and mtDNA release (Figure 5) [122]. This ANT1–SHP2 axis forms a negative feedback loop that restrains excessive inflammasome activation.

Recent data link ANT1 to cGAS–STING activating mtDNA release and ANT-dependent permeability control to innate immune polarization. Limiting ANT1 activity reduces mtDNA leakage and inflammatory signaling, extending ANT function to mitochondrial DAMP regulation [123].

Viewed in aggregate, cytokine networks do not merely regulate ANT but use ANT-dependent mitochondrial responses as an amplification or braking module within inflammatory signaling circuits. While cytokine-dependent regulation and inflammasome control illustrate how ANT-dependent mitochondrial dysfunction is converted into inflammatory signaling, mitochondrial RNA export represents a more direct route by which mitochondrial stress is exposed to cytosolic innate immune sensors, as described in Section 6.4.

### 6.4. ANT as a Mitochondrial RNA Translocon

**Figure 6:**
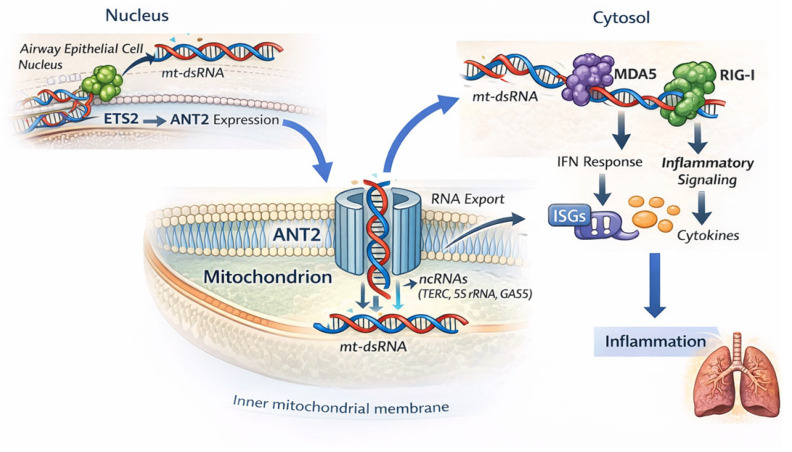
ANT in Mitochondrial RNA Export and RNA-Linked Immune Signaling The schematic illustrates the ETS2–ANT2–mt-dsRNA signaling axis linking mitochondrial RNA export to innate immune activation and airway inflammation. In airway epithelial cell nuclei, the transcription factor ETS2 binds to the ANT2 promoter and enhances ANT2 expression. Mitochondrial double-stranded RNA (mt-dsRNA) generated in the matrix is transported across the inner mitochondrial membrane by ANT2, which is proposed to function as an RNA translocon independently of its classical ADP/ATP carrier activity. ANT2 is proposed to translocate selected nuclear-encoded non-coding RNAs (e.g., TERC, 5S rRNA, GAS5). Exported mt-dsRNA accumulates in the cytosol, where it is sensed by the pattern-recognition receptors MDA5 and RIG-I, triggering interferon-stimulated gene (ISG) expression and pro-inflammatory cytokine production. In airway epithelial cells, increased ANT2 activity enhances mt-dsRNA release and promotes inflammatory responses associated with allergic airway disease. Schematic illustration based on published literature cited in the text.

Beyond mtDNA release, mitochondrial RNA has emerged as an ANT2-linked danger signal, although the precise transport mechanism and structural basis remain under active investigation. Recent studies suggest that ANT2 may participate in stress-associated mitochondrial RNA export, including double-stranded RNA (dsRNA), into the cytosol (Figure 6) [124,125]. This process is stress-responsive and associated with changes in membrane potential and ANT2 modification state, and appears mechanistically distinct from passive leakage during permeability transition.

Exported mtRNA activates cytosolic RNA sensors such as RIG-I and MDA5 and triggers type I interferon programs, linking mitochondrial dysfunction to innate immune signaling. In epithelial and immune cells, ANT2-dependent mtRNA export contributes to sustained interferon responses under conditions of metabolic or inflammatory stress.

A defined in vivo example is provided in allergic airway inflammation [126]. The transcription factor ETS2 is upregulated in airway epithelium and directly induces ANT2 expression. Increased ANT2 levels enhance cytosolic accumulation of mitochondrial RNA and dsRNA and amplify downstream cytokine responses. These pro-inflammatory effects are lost in ANT2-deficient models, supporting ANT2-dependent mtRNA export as a causal contributor linking mitochondrial stress to epithelial immune activation.

ANT2-mediated RNA export operates alongside other mitochondrial nucleic acid release routes—including defective PNPT1/SUV3-dependent RNA degradation, BAX/BAK macropores, VDAC oligomerization, and mitochondria-derived vesicles—and is functionally connected to permeability and quality-control pathways [127,128,129]. Together, these observations support a role for ANT2 in regulating mitochondrial RNA release during stress. Thereby, it links bioenergetic dysfunction to cytosolic RNA sensing and interferon responses. More broadly, these pathways intersect with immunometabolic programs, indicating that ANT-dependent stress signaling extends beyond epithelial and inflammatory responses to the metabolic specialization of immune cells, as discussed in Section 6.5.

### 6.5. ANT in Immune Cell Metabolism and Polarization

ANT’s immunological relevance is evident in immune cell metabolism. In T cells, ANT2 functions as an immunometabolic checkpoint. T cell–specific deletion of ANT2 uncouples activation from classical oxidative phosphorylation requirements, limits ATP synthase activity and NAD^+^ regeneration, yet paradoxically enhances proliferation and effector function [130]. ANT2-deficient T cells increase mitochondrial biogenesis and exhibit improved antitumor immunity in vivo.

In macrophages, ANT1 supports oxidative metabolism, mitophagy, and polarization toward an anti-inflammatory M2 phenotype. In M2-polarized macrophages, CD147 translocates to mitochondria and interacts with ANT1, enhancing PINK1/Parkin-dependent mitophagy and respiratory chain integrity [131]. Disruption of this axis impairs mitophagy, attenuates M2 polarization, and reduces pathological airway remodeling. Recent in vivo and cell-based data support the role of ANT1-dependent mitochondrial regulation in macrophage polarization within ischemic tissue [100]. Cardiac ANT1 overexpression is associated with reduced pro-inflammatory cytokine expression, decreased M1 prevalence, and increased M2 macrophage infiltration, together with preserved mitochondrial integrity and lower oxidative stress.

Complementary data indicate an isoform- and context-dependent pattern. In metabolically stressed adipose tissue macrophages, free fatty acids promote ANT2-linked mitochondrial stress signaling via mPTP-associated ROS with downstream HIF-1α and NF-κB activation, whereas myeloid ANT2 deficiency reduces monocyte recruitment and pro-inflammatory macrophage activation and improves systemic insulin sensitivity and glucose tolerance [132]. Thus, ANT1-associated mitochondrial stabilization tends to favor anti-inflammatory polarization, while ANT2-dependent stress signaling in metabolically challenged myeloid cells supports pro-inflammatory programs.

ANT1-dependent effects also extend beyond immune cells. Cardiomyocyte-specific ANT1 overexpression generates a protective secretome enriched in VEGF and HSP27, which improves mitochondrial function, suppresses apoptosis, and promotes anti-inflammatory macrophage responses in surrounding tissue [100]. Together, these findings establish ANT as a central immunometabolic regulator that integrates mitochondrial energy exchange with immune activation, polarization, and tissue–immune crosstalk. Once ANT-dependent mitochondrial states are linked to intracellular signaling and immune cell behavior, the next conceptual step is to consider how these signals extend beyond the individual cell. ANT-associated mechanisms at the plasma membrane and in extracellular vesicles suggest that mitochondrial state information can also be communicated at the tissue level.

## 7. ANT Beyond Mitochondria: Coupling Mitochondrial Function to Intercellular Communication

ANT-dependent mitochondrial information can also be transmitted beyond the cell to shape tissue-level and intercellular communication. A growing body of evidence demonstrates that ANT can operate outside its canonical mitochondrial context, either through ectopic localization at the plasma membrane or via incorporation into extracellular vesicles. In these settings, ANT enables the controlled export of energetic and stress-related signals, allowing mitochondrial function to influence neighboring cells and the tissue microenvironment.

### 7.1. ANT at the Plasma Membrane

Although ANT was originally defined as an inner mitochondrial membrane carrier, multiple studies report ectopic ANT localization at the plasma membrane in diverse mammalian cell types, including fibroblasts, hepatocytes, cancer cells, and neurons [133,134,135,136]. Surface biotinylation, antibody accessibility, and membrane fractionation approaches support that this pool is not solely due to mitochondrial contamination. In these settings, ANT contributes to extracellular nucleotide handling and local energy-dependent signaling.

At the cell surface, ANT has been linked to ATP-dependent control of migration and matrix remodeling. In tumor cells, plasma membrane-associated ANT2 interacts with MT1-MMP and is proposed to support pericellular proteolysis and invasion [135]. In developing neurons, surface-localized ANT1 and ANT2 participate in SRC family kinase-dependent signaling downstream of adhesion molecules, promoting transient ATP release and activity-dependent neurite growth [136].

In metabolic and vascular contexts, plasma membrane-associated ANT contributes to extracellular nucleotide balance and receptor-linked transport processes. In hepatocytes, ANT associates with ecto-F1-ATPase–containing complexes involved in HDL uptake, and pharmacological ANT modulation alters extracellular ADP/ATP ratios and lipoprotein internalization [134]. In human bronchial epithelial cells, ANT supports airway hydration and preserves ciliary beating following exposure to environmental stressors such as cigarette smoke, thereby sustaining mucociliary clearance [137]. Finally, in erythrocytes, ANT forms supramolecular complexes with VDAC and TSPO2 that support regulated ATP release and purinergic vascular signaling [138].

Ectopic ANT localization is also observed in intracellular pathogens, where ANT homologs mediate host-derived ATP uptake and extracellular nucleotide sensing, supporting pathogen survival under energy-limited conditions [139].

In sum, ectopic ANT localization extends nucleotide carrier function into the extracellular signaling space, where it contributes to purinergic control and energy-dependent cell–environment interactions.

### 7.2. ANT in Extracellular Vesicles

ANT also contributes to intercellular communication through incorporation into extracellular vesicles (EVs), particularly exosomes derived from multivesicular bodies. EVs transfer proteins, lipids, and nucleic acids between cells and thereby propagate metabolic and stress signals. Proteomic studies have detected ANT isoforms, including ANT1, ANT2, and ANT3, in EVs from cancer cells, immune cells, and stressed tissues [140,141,142]. These vesicles are distinct from mitochondria-derived vesicles (MDVs), which bud directly from mitochondria and selectively package mitochondrial components [143].

ANT-containing EVs have been proposed to convey aspects of mitochondrial functional state to recipient cells. Secretomes enriched in EVs from ANT1-overexpressing cardiomyocytes enhance survival of ischemic cardiomyocytes and endothelial cells and reduce pro-inflammatory activation in macrophages, demonstrating paracrine transfer of ANT-linked mitochondrial adaptation [100].

Conversely, EV-mediated delivery of ANT-modifying factors can influence ANT-dependent functions in recipient cells. For example, exosomal transfer of the deacetylase SIRT2 promotes deacetylation of ANT1/2 and enhances ATP production in axons [144]. EV-associated ANT and ANT-modifying factors therefore represent a vesicle-based extension of mitochondrial regulation, enabling ANT-dependent metabolic and permeability states to influence neighboring cells.

## 8. Conclusions

**Figure 7:**
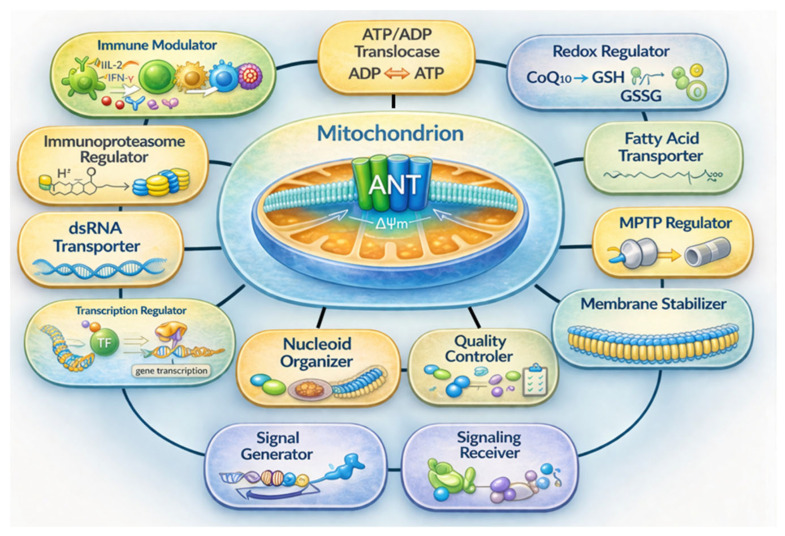
ANT as a multifunctional protein. The schematic integrates the diverse functions attributed to ANT, including nucleotide exchange, redox regulation, membrane stabilization, permeability control, nucleic acid-linked signaling, and communication across cellular compartments. Together, these functions position ANT as a context-dependent regulatory hub that stabilizes mitochondrial homeostasis under basal conditions and modulates adaptive or stress-associated responses when mitochondrial function is challenged.

Across the mechanisms discussed here, ANT emerges as a remarkably versatile mitochondrial protein. Its functions as a nucleotide, fatty acid, and dsRNA transporter, as well as a regulator, organizer, and signal sender and receiver, enabling it to influence a broad range of intracellular and extracellular processes (Figure 7). In this framework, ANT links these functions to bioenergetic control, quality maintenance, and cellular communication by modulating permeability transition sensitivity, protein import dynamics, and mitochondrial stress signaling. Through these activities, ANT helps determine whether mitochondrial perturbations are buffered, redirected into repair pathways, or propagated as adaptive or inflammatory signals.

The strength of mechanistic support varies across these domains. While nucleotide exchange and permeability regulation are firmly established, roles in mitochondrial RNA signaling, import complex interactions, and intercellular communication remain less structurally defined. In many systems, isoforms have not been examined in parallel, limiting definitive conclusions regarding intrinsic isoform specialization. At the same time, the available evidence indicates that ANT isoforms are not merely interchangeable carriers, but are deployed in distinct physiological and stress-responsive contexts, with overlapping yet biased functions across tissues and disease settings. A detailed process-oriented summary of these isoform-associated functional contexts is provided in Supplementary Table S1.

Functionally, ANT operates within feedback-organized signaling circuits: upstream kinase and cytokine pathways regulate ANT activity, whereas ANT-dependent changes in membrane potential, redox balance, and nucleic acid exposure feed back into cytosolic and nuclear programs. Because ANT integrates essential bioenergetic exchange with stress signaling control, future therapeutic strategies will likely require selective modulation of regulatory interactions and response thresholds rather than global transport inhibition.

Framing ANT as a modulator of mitochondrial bioenergetics, quality control, and cellular communication provides a coherent model that reconciles established carrier functions with emerging signaling roles while explicitly acknowledging current mechanistic limitations.

## Figures and Tables

**Table 1:** Regulatory miRNAs and cytokines targeting ANT isoform expression.

Effector	ANT Isoform	Mechanism of Action
miR-2861	ANT1	Direct binding to ANT1 coding sequence; suppresses ANT1 expression and sensitizes cardiomyocytes to necrotic cell death under oxidative stress
miR-29b	ANT2	Physical association with ANT2; co-localizes in perinuclear clusters and regulates nuclear morphology and mitotic progression
miR-636	ANT2	Induced upon ANT2 suppression; targets Ras and inhibits proliferation in hepatocellular carcinoma
miR-19a	ANT2	Reduced after ANT2 knockdown; modulates SOCS1/STAT3 signaling and oncogenic pathways
miR-96	ANT2	Downregulated upon ANT2 inhibition; contributes to suppression of tumor-promoting signaling
miR-21	ANT2	Reduced after ANT2 knockdown; restores SOCS1 expression and attenuates PI3K/AKT signaling
miR-221/222	ANT2	Downregulated after ANT2 suppression; limits STAT3-driven oncogenic signaling
miR-137	ANT2	Restoration reverses ANT2-driven cancer stemness and metastasis in HCC models
hcmv-miR-UL36-5p	ANT3	Viral miRNA suppresses ANT3 expression, inhibits apoptosis, and promotes viral persistence
TNFα	ANT1	NF-κB–dependent transcriptional repression; promotes ΔΨm loss, ROS production, and necrotic cell death
IL-6	ANT1	Suppresses ANT1 expression via inflammatory signaling pathways
IL-1β	ANT1	Downregulates ANT1 expression and enhances mitochondrial dysfunction
Interferons (type I)	ANT1	Repress ANT1 expression through inflammatory transcriptional programs
IL-4	ANT1	Induces ANT1 expression via tyrosine kinase, PI3K/AKT, ERK, and NF-κB signaling
IL-4	ANT3	Induces ANT3 expression in human T cells and promotes anti-apoptotic signaling
IFN-γ	ANT3	STAT1-dependent induction of ANT3; antagonized by IL-4/STAT6 signaling
TGF-β	ANT2	NF1/Smad4 repressor complex binds ANT2 promoter and suppresses transcription in senescent cells

## Data Availability

No new data were created or analyzed in this study.

## Supplementary Materials

The following supporting information can be downloaded at: https://www.mdpi.com/article/10.3390/cells15070646/s1. Table S1. Functional Roles of ANT Isoforms Across Cellular Processes.

## Abbreviations

AKT	Protein kinase B
ANT	Adenine nucleotide translocase
AP1	Activator protein 1
ATP	Adenosine triphosphate
BAK	BCL2 antagonist/killer
BAX	BCL2-associated X protein
CD147	Cluster of differentiation 147
cGAS	Cyclic GMP–AMP synthase
CypD	Cyclophilin D
DAMP	Damage-associated molecular pattern
DRP1	Dynamin-related protein 1
dsRNA	Double-stranded RNA
E2F6	E2F transcription factor 6
ERK1/2	Extracellular signal-regulated kinase 1/2
ERα	Estrogen receptor alpha
ETS2	ETS proto-oncogene 2 transcription factor
EV	Extracellular vesicle
F1FO	F1FO ATP synthase
FA	Fatty acid
GAS5	Growth arrest-specific transcript 5
GRBOX	Glycolysis-regulated box
GSK-3β	Glycogen synthase kinase 3 beta
GSNOR	S-nitrosoglutathione reductase
HDL	High-density lipoprotein
HER2	Human epidermal growth factor receptor 2
HIF-1α	Hypoxia-inducible factor 1 alpha
HSP27	Heat shock protein 27
HSP90	Heat shock protein 90
I/R	Ischemia/reperfusion
IFN	Interferon
IFN-γ	Interferon gamma
IL	Interleukin
IMM	Inner mitochondrial membrane
ISG	Interferon-stimulated gene
LC3	Microtubule-associated protein 1 light chain 3
M1	Classically activated macrophage phenotype
M2	Alternatively activated macrophage phenotype
MDA5	Melanoma differentiation-associated protein 5
MDV	Mitochondria-derived vesicle
MeCP2	Methyl-CpG-binding protein 2
MFN1/2	Mitofusin 1 and 2
MFN2	Mitofusin 2
miRNA	MicroRNA
mPTP	Mitochondrial permeability transition pore
mRNA	Messenger RNA
MT1-MMP	Membrane type 1 matrix metalloproteinase
mtDNA	Mitochondrial DNA
mtRNA	Mitochondrial RNA
NDPK-D	Nucleoside diphosphate kinase D
NFAT	Nuclear factor of activated T cells
NF-κB	Nuclear factor kappa B
NLRP3	NLR family pyrin domain-containing 3
NME4	Nucleoside diphosphate kinase 4
OMM	Outer mitochondrial membrane
OPA1	Optic atrophy protein 1
OXBOX	Oxidative metabolism box
OXPHOS	Oxidative phosphorylation
PAK6	p21-activated kinase 6
PHB1	Prohibitin 1
PHB2	Prohibitin 2
PI3K	Phosphoinositide 3-kinase
PINK1	PTEN-induced kinase 1
PNPT1	Polynucleotide phosphorylase 1
PTM	Post-translational modification
RB/E2F	Retinoblastoma/E2F complex
REBOX	Respiratory element box
RIG-I	Retinoic acid-inducible gene I
ROS	Reactive oxygen species
RyR2	Ryanodine receptor 2
SERCA2a	Sarcoplasmic/endoplasmic reticulum Ca^2+^-ATPase 2a
SHP2	SRC homology region 2-containing protein tyrosine phosphatase 2
SIRT4	Sirtuin 4
Smad	Small mothers against decapentaplegic protein
SOCS1	Suppressor of cytokine signaling 1
SP1	Specificity protein 1
SRC	SRC family kinase
STAT	Signal transducer and activator of transcription
STAT1	Signal transducer and activator of transcription 1
STAT3	Signal transducer and activator of transcription 3
STING	Stimulator of interferon genes
SUV3	ATP-dependent RNA helicase SUV3
TERC	Telomerase RNA component
TFAM	Mitochondrial transcription factor A
TGF-β	Transforming growth factor beta
TIMM	Translocase of the inner mitochondrial membrane
TIMM22	TIMM22 translocase complex
TIMM23	TIMM23 translocase complex
TIMM44	TIMM44 inner membrane import component
TLR4	Toll-like receptor 4
TNFα	Tumor necrosis factor alpha
TOMM	Translocase of the outer mitochondrial membrane
TSPO2	Translocator protein 2
UCP	Uncoupling protein
UCP3	Uncoupling protein 3
USP34	Ubiquitin-specific peptidase 34
VDAC	Voltage-dependent anion channel
VEGF	Vascular endothelial growth factor
YY1	Yin Yang 1
ZNF555	Zinc finger protein 555
ΔΨm	Mitochondrial membrane potential

## References

[1] Klingenberg M. (2008). The ADP and ATP transport in mitochondria and its carrier. Biochim. Biophys. Acta.

[2] Pebay-Peyroula E., Dahout-Gonzalez C., Kahn R., Trézéguet V., Lauquin G.J., Brandolin G. (2003). Structure of mitochondrial ADP/ATP carrier in complex with carboxyatractyloside. Nature.

[3] Cimadamore-Werthein C., King M.S., Lacabanne D., Pyrihova E., Jaiquel Baron S., Kunji E.R. (2024). Human mitochondrial carriers of the SLC25 family function as monomers exchanging substrates with a ping-pong kinetic mechanism. EMBO J..

[4] Mishra G., Coyne L.P., Chen X.J. (2023). Adenine nucleotide carrier protein dysfunction in human disease. IUBMB Life.

[5] Chen Y., Wu L., Liu J., Ma L., Zhang W. (2023). Adenine nucleotide translocase: Current knowledge in post-translational modifications, regulations and pathological implications for human diseases. FASEB J..

[6] Frigo E., Tommasin L., Lippe G., Carraro M., Bernardi P. (2023). The Haves and Have-Nots: The Mitochondrial Permeability Transition Pore across Species. Cells.

[7] Doerner A., Pauschinger M., Badorff A., Noutsias M., Giessen S., Schulze K., Bilger J., Rauch U., Schultheiss H.P. (1997). Tissue-specific transcription pattern of the adenine nucleotide translocase isoforms in humans. FEBS Lett..

[8] Traba J., Satrustegui J., del Arco A. (2011). Adenine nucleotide transporters in organelles: Novel genes and functions. Cell Mol. Life Sci..

[9] Li K., Hodge J.A., Wallace D.C. (1990). OXBOX, a positive transcriptional element of the heart-skeletal muscle ADP/ATP translocator gene. J. Biol. Chem..

[10] Chung A.B., Stepien G., Haraguchi Y., Li K., Wallace D.C. (1992). Transcriptional control of nuclear genes for the mitochondrial muscle ADP/ATP translocator and the ATP synthase beta subunit. Multiple factors interact with the OXBOX/REBOX promoter sequences. J. Biol. Chem..

[11] Zhang C., Jiang H., Wang P., Liu H., Sun X. (2017). Transcription factor NF-kappa B represses ANT1 transcription and leads to mitochondrial dysfunctions. Sci. Rep..

[12] Forlani G., Giarda E., Ala U., Di Cunto F., Salani M., Tupler R., Kilstrup-Nielsen C., Landsberger N. (2010). The MeCP2/YY1 interaction regulates ANT1 expression at 4q35: Novel hints for Rett syndrome pathogenesis. Hum. Mol. Genet..

[13] Kim E., Rich J., Karoutas A., Tarlykov P., Cochet E., Malysheva D., Mamchaoui K., Ogryzko V., Pirozhkova I. (2015). ZNF555 protein binds to transcriptional activator site of 4qA allele and ANT1: Potential implication in Facioscapulohumeral dystrophy. Nucleic Acids Res..

[14] Law A.K., Gupta D., Levy S., Wallace D.C., McKeon R.J., Buck C.R. (2004). TGF-beta1 induction of the adenine nucleotide translocator 1 in astrocytes occurs through Smads and Sp1 transcription factors. BMC Neurosci..

[15] Rottensteiner H., Palmieri L., Hartig A., Hamilton B., Ruis H., Erdmann R., Gurvitz A. (2002). The peroxisomal transporter gene ANT1 is regulated by a deviant oleate response element (ORE): Characterization of the signal for fatty acid induction. Biochem. J..

[16] Giraud S., Bonod-Bidaud C., Wesolowski-Louvel M., Stepien G. (1998). Expression of human ANT2 gene in highly proliferative cells: GRBOX, a new transcriptional element, is involved in the regulation of glycolytic ATP import into mitochondria. J. Mol. Biol..

[17] Slim R., Levilliers J., Lüdecke H.J., Claussen U., Nguyen V.C., Gough N.M., Horsthemke B., Petit C. (1993). A human pseudoautosomal gene encodes the ANT3 ADP/ATP translocase and escapes X-inactivation. Genomics.

[18] Dupont P.Y., Stepien G. (2011). Computational analysis of the transcriptional regulation of the adenine nucleotide translocator isoform 4 gene and its role in spermatozoid glycolytic metabolism. Gene.

[19] Rodić N., Oka M., Hamazaki T., Murawski M.R., Jorgensen M., Maatouk D.M., Resnick J.L., Li E., Terada N. (2005). DNA methylation is required for silencing of ant4, an adenine nucleotide translocase selectively expressed in mouse embryonic stem cells and germ cells. Stem Cells.

[20] Kehoe S.M., Oka M., Hankowski K.E., Reichert N., Garcia S., McCarrey J.R., Gaubatz S., Terada N. (2008). A conserved E2F6-binding element in murine meiosis-specific gene promoters. Biol. Reprod..

[21] Graham B.H., Waymire K.G., Cottrell B., Trounce I.A., MacGregor G.R., Wallace D.C. (1997). A mouse model for mitochondrial myopathy and cardiomyopathy resulting from a deficiency in the heart/muscle isoform of the adenine nucleotide translocator. Nat. Genet..

[22] Kokoszka J.E., Waymire K.G., Flierl A., Sweeney K.M., Angelin A., MacGregor G.R., Wallace D.C. (2016). Deficiency in the mouse mitochondrial adenine nucleotide translocator isoform 2 gene is associated with cardiac noncompaction. Biochim. Biophys. Acta.

[23] Jang J.Y., Lee C.E. (2003). Mitochondrial adenine nucleotide translocator 3 is regulated by IL-4 and IFN-gamma via STAT-dependent pathways. Cell Immunol..

[24] Brower J.V., Lim C.H., Jorgensen M., Oh S.P., Terada N. (2009). Adenine nucleotide translocase 4 deficiency leads to early meiotic arrest of murine male germ cells. Reproduction.

[25] Stepien G., Torroni A., Chung A.B., Hodge J.A., Wallace D.C. (1992). Differential expression of adenine nucleotide translocator isoforms in mammalian tissues and during muscle cell differentiation. J. Biol. Chem..

[26] Gavaldà-Navarro A., Mampel T., Viñas O. (2016). Changes in the expression of the human adenine nucleotide translocase isoforms condition cellular metabolic/proliferative status. Open Biol..

[27] Pan T., Yang B., Yao S., Wang R., Zhu Y. (2024). Exploring the multifaceted role of adenosine nucleotide translocase 2 in cellular and disease processes: A comprehensive review. Life Sci..

[28] Dorner A., Giessen S., Gaub R., Grosse Siestrup H., Schwimmbeck P.L., Hetzer R., Poller W., Schultheiss H.P. (2006). An isoform shift in the cardiac adenine nucleotide translocase expression alters the kinetic properties of the carrier in dilated cardiomyopathy. Eur. J. Heart Fail..

[29] Flierl A., Schriner S.E., Hancock S., Coskun P.E., Wallace D.C. (2022). The mitochondrial adenine nucleotide transporters in myogenesis. Free Radic. Biol. Med..

[30] Gouriou Y., Alam M.R., Harhous Z., Crola Da Silva C., Baetz D.B., Badawi S., Lefai E., Rieusset J., Durand A., Harisseh R. (2020). ANT2-Mediated ATP Import into Mitochondria Protects against Hypoxia Lethal Injury. Cells.

[31] Vyssokikh M.Y., Katz A., Rueck A., Wuensch C., Dörner A., Zorov D.B., Brdiczka D. (2001). Adenine nucleotide translocator isoforms 1 and 2 are differently distributed in the mitochondrial inner membrane and have distinct affinities to cyclophilin D. Biochem. J..

[32] Letts J.A., Fiedorczuk K., Sazanov L.A. (2016). The architecture of respiratory supercomplexes. Nature.

[33] Nesterov S., Chesnokov Y., Kamyshinsky R., Panteleeva A., Lyamzaev K., Vasilov R., Yaguzhinsky L. (2021). Ordered Clusters of the Complete Oxidative Phosphorylation System in Cardiac Mitochondria. Int. J. Mol. Sci..

[34] Lu Y.W., Acoba M.G., Selvaraju K., Huang T.C., Nirujogi R.S., Sathe G., Pandey A., Claypool S.M. (2017). Human adenine nucleotide translocases physically and functionally interact with respirasomes. Mol. Biol. Cell.

[35] M. Parodi-Rullán R., Chapa-Dubocq X., Guzmán-Hernández R., Jang S., A. Torres-Ramos C., Ayala-Peña S., Javadov S. (2019). The Role of Adenine Nucleotide Translocase in the Assembly of Respiratory Supercomplexes in Cardiac Cells. Cells.

[36] Roussel D., Roussel N., Voituron Y., Rey B. (2024). Liver mitochondrial coupling efficiency and its relationship to oxidative capacity and adenine nucleotide translocase content: A comparative study among crocodiles, birds and mammals. Mitochondrion.

[37] Belosludtseva N.V., Ilzorkina A.I., Serov D.A., Dubinin M.V., Talanov E.Y., Karagyaur M.N., Primak A.L., Liu J., Belosludtsev K.N. (2024). ANT-Mediated Inhibition of the Permeability Transition Pore Alleviates Palmitate-Induced Mitochondrial Dysfunction and Lipotoxicity. Biomolecules.

[38] Kreiter J., Rupprecht A., Škulj S., Brkljača Z., Žuna K., Knyazev D.G., Bardakji S., Vazdar M., Pohl E.E. (2021). ANT1 Activation and Inhibition Patterns Support the Fatty Acid Cycling Mechanism for Proton Transport. Int. J. Mol. Sci..

[39] Schönfeld P., Wojtczak L. (2016). Short- and medium-chain fatty acids in energy metabolism: The cellular perspective. J. Lipid Res..

[40] Bevilacqua L., Seifert E.L., Estey C., Gerrits M.F., Harper M.E. (2010). Absence of uncoupling protein-3 leads to greater activation of an adenine nucleotide translocase-mediated proton conductance in skeletal muscle mitochondria from calorie restricted mice. Biochim. Biophys. Acta.

[41] Friederich M., Nordquist L., Olerud J., Johansson M., Hansell P., Palm F. (2009). Identification and distribution of uncoupling protein isoforms in the normal and diabetic rat kidney. Adv. Exp. Med. Biol..

[42] Brand M.D., Pakay J.L., Ocloo A., Kokoszka J., Wallace D.C., Brookes P.S., Cornwall E.J. (2005). The basal proton conductance of mitochondria depends on adenine nucleotide translocase content. Biochem. J..

[43] Schiffer T.A., Löf L., Gallini R., Kamali-Moghaddam M., Carlström M., Palm F. (2022). Mitochondrial Respiration-Dependent ANT2-UCP2 Interaction. Front. Physiol..

[44] Chen Y., Liu J., Zheng Y., Wang J., Wang Z., Gu S., Tan J., Jing Q., Yang H. (2015). Uncoupling protein 3 mediates H_2_O_2_ preconditioning-afforded cardioprotection through the inhibition of MPTP opening. Cardiovasc. Res..

[45] Schulz R., Schluter K.D. (2023). Importance of Mitochondria in Cardiac Pathologies: Focus on Uncoupling Proteins and Monoamine Oxidases. Int. J. Mol. Sci..

[46] Dörner A., Lynetskiy O., Euler G., Landmesser U., Schlüter K.D., Heger J. (2021). Mitochondria Isolated from Hearts Subjected to Ischemia/Reperfusion Benefit from Adenine Nucleotide Translocase 1 Overexpression. Membranes.

[47] Chen J.J., Bertrand H., Yu B.P. (1995). Inhibition of adenine nucleotide translocator by lipid peroxidation products. Free Radic. Biol. Med..

[48] Eaton P., Li J.M., Hearse D.J., Shattock M.J. (1999). Formation of 4-hydroxy-2-nonenal-modified proteins in ischemic rat heart. Am. J. Physiol..

[49] Zwizinski C.W., Schmid H.H. (1992). Peroxidative damage to cardiac mitochondria: Identification and purification of modified adenine nucleotide translocase. Arch. Biochem. Biophys..

[50] Vieira H.L., Belzacq A.S., Haouzi D., Bernassola F., Cohen I., Jacotot E., Ferri K.F., El Hamel C., Bartle L.M., Melino G. (2001). The adenine nucleotide translocator: A target of nitric oxide, peroxynitrite, and 4-hydroxynonenal. Oncogene.

[51] Klumpe I., Savvatis K., Westermann D., Tschope C., Rauch U., Landmesser U., Schultheiss H.P., Dorner A. (2016). Transgenic overexpression of adenine nucleotide translocase 1 protects ischemic hearts against oxidative stress. J. Mol. Med..

[52] Yan L.J., Sohal R.S. (1998). Mitochondrial adenine nucleotide translocase is modified oxidatively during aging. Proc. Natl. Acad. Sci. USA.

[53] Jovanović O., Chekashkina K., Škulj S., Žuna K., Vazdar M., Bashkirov P.V., Pohl E.E. (2022). Membrane Lipid Reshaping Underlies Oxidative Stress Sensing by the Mitochondrial Proteins UCP1 and ANT1. Antioxidants.

[54] Brustovetsky N. (2020). The Role of Adenine Nucleotide Translocase in the Mitochondrial Permeability Transition. Cells.

[55] Winter J., Klumpe I., Heger J., Rauch U., Schultheiss H.P., Landmesser U., Dorner A. (2016). Adenine nucleotide translocase 1 overexpression protects cardiomyocytes against hypoxia via increased ERK1/2 and AKT activation. Cell Signal.

[56] Liu J., Ding W., Chen Q., Peng Y., Kong Y., Ma L., Zhang W. (2025). Adenine Nucleotide Translocase 1 Promotes Functional Integrity of Mitochondria via Activating DDIT3-CytC Pathway and Intensifying Actin Filament Structures. Mol. Neurobiol..

[57] Beutner G., Rück A., Riede B., Brdiczka D. (1998). Complexes between porin, hexokinase, mitochondrial creatine kinase and adenylate translocator display properties of the permeability transition pore. Implication for regulation of permeability transition by the kinases. Biochim. Biophys. Acta.

[58] Brustovetsky N., Tropschug M., Heimpel S., Heidkämper D., Klingenberg M. (2002). A large Ca^2+^-dependent channel formed by recombinant ADP/ATP carrier from Neurospora crassa resembles the mitochondrial permeability transition pore. Biochemistry.

[59] Bernardi P. (2020). Mechanisms for Ca^2+^-dependent permeability transition in mitochondria. Proc. Natl. Acad. Sci. USA.

[60] McEnery M.W., Snowman A.M., Trifiletti R.R., Snyder S.H. (1992). Isolation of the mitochondrial benzodiazepine receptor: Association with the voltage-dependent anion channel and the adenine nucleotide carrier. Proc. Natl. Acad. Sci. USA.

[61] Kokoszka J.E., Waymire K.G., Levy S.E., Sligh J.E., Cai J., Jones D.P., MacGregor G.R., Wallace D.C. (2004). The ADP/ATP translocator is not essential for the mitochondrial permeability transition pore. Nature.

[62] Karch J., Bround M.J., Khalil H., Sargent M.A., Latchman N., Terada N., Peixoto P.M., Molkentin J.D. (2019). Inhibition of mitochondrial permeability transition by deletion of the ANT family and CypD. Sci. Adv..

[63] Bernardi P., Gerle C., Halestrap A.P., Jonas E.A., Karch J., Mnatsakanyan N., Pavlov E., Sheu S.S., Soukas A.A. (2023). Identity, structure, and function of the mitochondrial permeability transition pore: Controversies, consensus, recent advances, and future directions. Cell Death Differ..

[64] Alavian K.N., Beutner G., Lazrove E., Sacchetti S., Park H.A., Licznerski P., Li H., Nabili P., Hockensmith K., Graham M. (2014). An uncoupling channel within the c-subunit ring of the F1FO ATP synthase is the mitochondrial permeability transition pore. Proc. Natl. Acad. Sci. USA.

[65] Pekson R., Liang F.G., Axelrod J.L., Lee J., Qin D., Wittig A.J.H., Paulino V.M., Zheng M., Peixoto P.M., Kitsis R.N. (2023). The mitochondrial ATP synthase is a negative regulator of the mitochondrial permeability transition pore. Proc. Natl. Acad. Sci. USA.

[66] Ban T., Ishihara T., Kohno H., Saita S., Ichimura A., Maenaka K., Oka T., Mihara K., Ishihara N. (2017). Molecular basis of selective mitochondrial fusion by heterotypic action between OPA1 and cardiolipin. Nat. Cell Biol..

[67] Chen H., Chan D.C. (2004). Mitochondrial dynamics in mammals. Curr. Top. Dev. Biol..

[68] Mishra P., Carelli V., Manfredi G., Chan D.C. (2014). Proteolytic cleavage of Opa1 stimulates mitochondrial inner membrane fusion and couples fusion to oxidative phosphorylation. Cell Metab..

[69] Schlattner U., Tokarska-Schlattner M., Epand R.M., Boissan M., Lacombe M.L., Kagan V.E. (2018). NME4/nucleoside diphosphate kinase D in cardiolipin signaling and mitophagy. Lab. Investig..

[70] Donnarumma E., Kohlhaas M., Vimont E., Kornobis E., Chaze T., Gianetto Q.G., Matondo M., Moya-Nilges M., Maack C., Wai T. (2022). Mitochondrial Fission Process 1 controls inner membrane integrity and protects against heart failure. Nat. Commun..

[71] Picca A., Mankowski R.T., Burman J.L., Donisi L., Kim J.S., Marzetti E., Leeuwenburgh C. (2018). Mitochondrial quality control mechanisms as molecular targets in cardiac ageing. Nat. Rev. Cardiol..

[72] Eldeeb M.A., Fallahi A., Soumbasis A., Bayne A.N., Trempe J.F., Fon E.A. (2024). Mitochondrial import stress and PINK1-mediated mitophagy: The role of the PINK1-TOMM-TIMM23 supercomplex. Autophagy.

[73] Pickles S., Vigie P., Youle R.J. (2018). Mitophagy and Quality Control Mechanisms in Mitochondrial Maintenance. Curr. Biol..

[74] Onishi M., Yamano K., Sato M., Matsuda N., Okamoto K. (2021). Molecular mechanisms and physiological functions of mitophagy. EMBO J..

[75] Hoshino A., Wang W.J., Wada S., McDermott-Roe C., Evans C.S., Gosis B., Morley M.P., Rathi K.S., Li J., Li K. (2019). The ADP/ATP translocase drives mitophagy independent of nucleotide exchange. Nature.

[76] Greene A.W., Grenier K., Aguileta M.A., Muise S., Farazifard R., Haque M.E., McBride H.M., Park D.S., Fon E.A. (2012). Mitochondrial processing peptidase regulates PINK1 processing, import and Parkin recruitment. EMBO Rep..

[77] Jiang S., Chen D., Sheng R., Yuan Q., Pan J., Guo Y. (2026). USP34 attenuates cartilage degradation in temporomandibular joint osteoarthritis by ANT1-mediated mitophagy. JBMR Plus.

[78] Tang X., Zhao S., Liu J., Liu X., Sha X., Huang C., Hu L., Sun S., Gao Y., Chen H. (2023). Mitochondrial GSNOR Alleviates Cardiac Dysfunction via ANT1 Denitrosylation. Circ. Res..

[79] Li N., Xu H., Liu X., Gao R., He J., Ding Y., Li F., Geng Y., Mu X., Chen X. (2022). Exposure to benzo(a)pyrene suppresses mitophagy via ANT1-PINK1-Parkin pathway in ovarian corpus luteum during early pregnancy. Sci. Total Environ..

[80] Wang N., Li W., Yang T., Li B., Meng C., Zhou X., Sun J., Yu K., Cui S., Cao R. (2025). Targeting CB1R Rewires Ca^2+^-Dependent Mitophagy to Promote Nerve Regeneration. Theranostics.

[81] Hu K., Lai Y., Zhou J., Hu C., Guo S., Zhang H., Wang G., Zhang Q., Gao X., Wang Z. (2025). Aberrant activation of adenine nucleotide translocase 3 promotes progression and chemoresistance in multiple myeloma dependent on PINK1 transport. Int. J. Biol. Sci..

[82] Ferramosca A., Zara V. (2013). Biogenesis of mitochondrial carrier proteins: Molecular mechanisms of import into mitochondria. Biochim. Biophys. Acta.

[83] Coyne L.P., Wang X., Song J., de Jong E., Schneider K., Massa P.T., Middleton F.A., Becker T., Chen X.J. (2023). Mitochondrial protein import clogging as a mechanism of disease. eLife.

[84] Fontanesi F., Palmieri L., Scarcia P., Lodi T., Donnini C., Limongelli A., Tiranti V., Zeviani M., Ferrero I., Viola A.M. (2004). Mutations in AAC2, equivalent to human adPEO-associated ANT1 mutations, lead to defective oxidative phosphorylation in Saccharomyces cerevisiae and affect mitochondrial DNA stability. Hum. Mol. Genet..

[85] Kaukonen J., Juselius J.K., Tiranti V., Kyttälä A., Zeviani M., Comi G.P., Keränen S., Peltonen L., Suomalainen A. (2000). Role of adenine nucleotide translocator 1 in mtDNA maintenance. Science.

[86] Palmieri L., Alberio S., Pisano I., Lodi T., Meznaric-Petrusa M., Zidar J., Santoro A., Scarcia P., Fontanesi F., Lamantea E. (2005). Complete loss-of-function of the heart/muscle-specific adenine nucleotide translocator is associated with mitochondrial myopathy and cardiomyopathy. Hum. Mol. Genet..

[87] Wang X., Salinas K., Zuo X., Kucejova B., Chen X.J. (2008). Dominant membrane uncoupling by mutant adenine nucleotide translocase in mitochondrial diseases. Hum. Mol. Genet..

[88] Esposito L.A., Melov S., Panov A., Cottrell B.A., Wallace D.C. (1999). Mitochondrial disease in mouse results in increased oxidative stress. Proc. Natl. Acad. Sci. USA.

[89] Lee S.R., Han J. (2017). Mitochondrial Nucleoid: Shield and Switch of the Mitochondrial Genome. Oxidative Med. Cell. Longev..

[90] Bogenhagen D.F., Wang Y., Shen E.L., Kobayashi R. (2003). Protein components of mitochondrial DNA nucleoids in higher eukaryotes. Mol. Cell Proteom..

[91] Kasashima K., Ohta E., Kagawa Y., Endo H. (2006). Mitochondrial functions and estrogen receptor-dependent nuclear translocation of pleiotropic human prohibitin 2. J. Biol. Chem..

[92] Kuramori C., Azuma M., Kume K., Kaneko Y., Inoue A., Yamaguchi Y., Kabe Y., Hosoya T., Kizaki M., Suematsu M. (2009). Capsaicin binds to prohibitin 2 and displaces it from the mitochondria to the nucleus. Biochem. Biophys. Res. Commun..

[93] Ito K., Nakazato T., Yamato K., Miyakawa Y., Yamada T., Hozumi N., Segawa K., Ikeda Y., Kizaki M. (2004). Induction of apoptosis in leukemic cells by homovanillic acid derivative, capsaicin, through oxidative stress: Implication of phosphorylation of p53 at Ser-15 residue by reactive oxygen species. Cancer Res..

[94] Alula K.M., Delgado-Deida Y., Callahan R., Till A., Underwood L., Thompson W.E., Souza R.F., Dassopoulos T., Onyiah J., Venuprasad K. (2023). Inner mitochondrial membrane protein Prohibitin 1 mediates Nix-induced, Parkin-independent mitophagy. Sci. Rep..

[95] Merkwirth C., Langer T. (2009). Prohibitin function within mitochondria: Essential roles for cell proliferation and cristae morphogenesis. Biochim. Biophys. Acta.

[96] Kobayashi H., Miura T., Ishida H., Miki T., Tanno M., Yano T., Sato T., Hotta H., Shimamoto K. (2008). Limitation of infarct size by erythropoietin is associated with translocation of Akt to the mitochondria after reperfusion. Clin. Exp. Pharmacol. Physiol..

[97] Das S., Wong R., Rajapakse N., Murphy E., Steenbergen C. (2008). Glycogen synthase kinase 3 inhibition slows mitochondrial adenine nucleotide transport and regulates voltage-dependent anion channel phosphorylation. Circ. Res..

[98] Zhai P., Sadoshima J. (2008). Overcoming an energy crisis?: An adaptive role of glycogen synthase kinase-3 inhibition in ischemia/reperfusion. Circ. Res..

[99] Jang J.Y., Jeon Y.K., Kim C.W. (2010). Degradation of HER2/neu by ANT2 shRNA suppresses migration and invasiveness of breast cancer cells. BMC Cancer.

[100] Yergoz F., Friebel J., Krankel N., Rauch-Kroehnert U., Schultheiss H.P., Landmesser U., Dorner A. (2021). Adenine Nucleotide Translocase 1 Expression Modulates the Immune Response in Ischemic Hearts. Cells.

[101] Feng J., Lucchinetti E., Enkavi G., Wang Y., Gehrig P., Roschitzki B., Schaub M.C., Tajkhorshid E., Zaugg K., Zaugg M. (2010). Tyrosine phosphorylation by Src within the cavity of the adenine nucleotide translocase 1 regulates ADP/ATP exchange in mitochondria. Am. J. Physiol. Cell Physiol..

[102] Hsu P.C., Yang C.T., Jablons D.M., You L. (2020). The Crosstalk between Src and Hippo/YAP Signaling Pathways in Non-Small Cell Lung Cancer (NSCLC). Cancers.

[103] Gavaldà-Navarro A., Villena J.A., Planavila A., Viñas O., Mampel T. (2014). Expression of adenine nucleotide translocase (ANT) isoform genes is controlled by PGC-1α through different transcription factors. J. Cell Physiol..

[104] Li T., Li Y., Liu T., Hu B., Li J., Liu C., Liu T., Li F. (2020). Mitochondrial PAK6 inhibits prostate cancer cell apoptosis via the PAK6-SIRT4-ANT2 complex. Theranostics.

[105] Ihara Y., Suzuki Y.J., Kitta K., Jones L.R., Ikeda T. (2002). Modulation of gene expression in transgenic mouse hearts overexpressing calsequestrin. Cell Calcium.

[106] Roussel J., Thireau J., Brenner C., Saint N., Scheuermann V., Lacampagne A., Le Guennec J.Y., Fauconnier J. (2015). Palmitoyl-carnitine increases RyR2 oxidation and sarcoplasmic reticulum Ca^2+^ leak in cardiomyocytes: Role of adenine nucleotide translocase. Biochim. Biophys. Acta.

[107] Yamaguchi N., Kagari T., Kasai M. (1999). Inhibition of the ryanodine receptor calcium channel in the sarcoplasmic reticulum of skeletal muscle by an ADP/ATP translocase inhibitor, atractyloside. Biochem. Biophys. Res. Commun..

[108] Vogelpohl I., Vetter R., Heger J., Ebermann L., Euler G., Schultheiss H.P., Dorner A. (2011). Transgenic overexpression of heart-specific adenine nucleotide translocase 1 positively affects contractile function in cardiomyocytes. Cell Physiol. Biochem..

[109] Walther T., Tschope C., Sterner-Kock A., Westermann D., Heringer-Walther S., Riad A., Nikolic A., Wang Y., Ebermann L., Siems W.E. (2007). Accelerated mitochondrial adenosine diphosphate/adenosine triphosphate transport improves hypertension-induced heart disease. Circulation.

[110] Wang Y., Ebermann L., Sterner-Kock A., Wika S., Schultheiss H.P., Dorner A., Walther T. (2009). Myocardial overexpression of adenine nucleotide translocase 1 ameliorates diabetic cardiomyopathy in mice. Exp. Physiol..

[111] Wang K., Long B., Li N., Li L., Liu C.Y., Dong Y.H., Gao J.N., Zhou L.Y., Wang C.Q., Li P.F. (2016). MicroRNA-2861 regulates programmed necrosis in cardiomyocyte by impairing adenine nucleotide translocase 1 expression. Free Radic. Biol. Med..

[112] Kriegel A.J., Terhune S.S., Greene A.S., Noon K.R., Pereckas M.S., Liang M. (2018). Isomer-specific effect of microRNA miR-29b on nuclear morphology. J. Biol. Chem..

[113] Jang J.Y., Lee Y.S., Jeon Y.K., Lee K., Jang J.J., Kim C.W. (2013). ANT2 suppression by shRNA restores miR-636 expression, thereby downregulating Ras and inhibiting tumorigenesis of hepatocellular carcinoma. Exp. Mol. Med..

[114] Jang J.Y., Jeon Y.K., Lee C.E., Kim C.W. (2013). ANT2 suppression by shRNA may be able to exert anticancer effects in HCC further by restoring SOCS1 expression. Int. J. Oncol..

[115] Baik S.H., Lee J., Lee Y.S., Jang J.Y., Kim C.W. (2016). ANT2 shRNA downregulates miR-19a and miR-96 through the PI3K/Akt pathway and suppresses tumor growth in hepatocellular carcinoma cells. Exp. Mol. Med..

[116] Jang J.Y., Kim Y.G., Nam S.J., Keam B., Kim T.M., Jeon Y.K., Kim C.W. (2016). Targeting Adenine Nucleotide Translocase-2 (ANT2) to Overcome Resistance to Epidermal Growth Factor Receptor Tyrosine Kinase Inhibitor in Non-Small Cell Lung Cancer. Mol. Cancer Ther..

[117] Lu A.Q., Lv B., Qiu F., Wang X.Y., Cao X.H. (2017). Upregulation of miR-137 reverses sorafenib resistance and cancer-initiating cell phenotypes by degrading ANT2 in hepatocellular carcinoma. Oncol. Rep..

[118] Guo X., Huang Y., Qi Y., Liu Z., Ma Y., Shao Y., Jiang S., Sun Z., Ruan Q. (2015). Human cytomegalovirus miR-UL36-5p inhibits apoptosis via downregulation of adenine nucleotide translocator 3 in cultured cells. Arch. Virol..

[119] Jang J.Y., Lee C.E. (2006). IL-4-induced upregulation of adenine nucleotide translocase 3 and its role in Th cell survival from apoptosis. Cell Immunol..

[120] Kretova M., Sabova L., Hodny Z., Bartek J., Kollarovic G., Nelson B.D., Hubackova S., Luciakova K. (2014). TGF-β/NF1/Smad4-mediated suppression of ANT2 contributes to oxidative stress in cellular senescence. Cell Signal.

[121] Heger J., Abdallah Y., Shahzad T., Klumpe I., Piper H.M., Schultheiss H.P., Schluter K.D., Schulz R., Euler G., Dorner A. (2012). Transgenic overexpression of the adenine nucleotide translocase 1 protects cardiomyocytes against TGFbeta1-induced apoptosis by stabilization of the mitochondrial permeability transition pore. J. Mol. Cell Cardiol..

[122] Guo W., Liu W., Chen Z., Gu Y., Peng S., Shen L., Shen Y., Wang X., Feng G.S., Sun Y. (2017). Tyrosine phosphatase SHP2 negatively regulates NLRP3 inflammasome activation via ANT1-dependent mitochondrial homeostasis. Nat. Commun..

[123] Zhao Y., Shi F., Hao H., Wang Z., Li J., Liu B., Niu Y., Wang W., Zhang Y. (2025). Hypertension Increases Susceptibility to Lead-Induced Microglial Polarization via ANT1-Mediated Mitochondrial DNA/cGAS/STING Signaling. Research.

[124] Wang P., Zhang L., Chen S., Li R., Liu P., Li X., Luo H., Huo Y., Zhang Z., Cai Y. (2024). ANT2 functions as a translocon for mitochondrial cross-membrane translocation of RNAs. Cell Res..

[125] Zhu H., Lin W., Lin A. (2024). ANT2: The first mammalian mitochondrial RNA transport translocon. Cell Res..

[126] Jiang H., Jiang Y., Dong R., Fu C.Y. (2025). ETS2 aggravate allergic airway inflammation by regulating ANT2-mediated cytosolic mitochondrial DsRNA levels. Respir. Res..

[127] Wang G., Chen H.W., Oktay Y., Zhang J., Allen E.L., Smith G.M., Fan K.C., Hong J.S., French S.W., McCaffery J.M. (2010). PNPASE regulates RNA import into mitochondria. Cell.

[128] Krieger M.R., Abrahamian M., He K.L., Atamdede S., Hakimjavadi H., Momcilovic M., Ostrow D., Maggo S.D., Tsang Y.P., Gai X. (2024). Trafficking of mitochondrial double-stranded RNA from mitochondria to the cytosol. Life Sci. Alliance.

[129] Belzacq A.S., Vieira H.L., Verrier F., Vandecasteele G., Cohen I., Prevost M.C., Larquet E., Pariselli F., Petit P.X., Kahn A. (2003). Bcl-2 and Bax modulate adenine nucleotide translocase activity. Cancer Res..

[130] Yosef O., Cohen-Daniel L., Shamriz O., Bar-On Z., Salaymeh W., Saragovi A., Abramovich I., Agranovich B., Lutz V., Tam J. (2025). Metabolic reprogramming driven by Ant2 deficiency augments T Cell function and anti-tumor immunity in mice. Nat. Commun..

[131] Zhu G., Yu H., Li X., Ye W., Chen X., Gu W. (2025). CD147 mitochondria translocation induced airway remodeling in asthmatic mouse models by regulating M2 macrophage polarization via ANT1-mediated mitophagy. Am. J. Physiol. Cell Physiol..

[132] Seo J.B., Riopel M., Cabrales P., Huh J.Y., Bandyopadhyay G.K., Andreyev A.Y., Murphy A.N., Beeman S.C., Smith G.I., Klein S. (2019). Knockdown of Ant2 Reduces Adipocyte Hypoxia and Improves Insulin Resistance in Obesity. Nat. Metab..

[133] Sigal C.T., Resh M.D. (1993). The ADP/ATP carrier is the 32-kilodalton receptor for an NH2-terminally myristylated src peptide but not for pp60src polypeptide. Mol. Cell Biol..

[134] Cardouat G., Duparc T., Fried S., Perret B., Najib S., Martinez L.O. (2017). Ectopic adenine nucleotide translocase activity controls extracellular ADP levels and regulates the F(1)-ATPase-mediated HDL endocytosis pathway on hepatocytes. Biochim. Biophys. Acta Mol. Cell Biol. Lipids.

[135] Radichev I.A., Remacle A.G., Sounni N.E., Shiryaev S.A., Rozanov D.V., Zhu W., Golubkova N.V., Postnova T.I., Golubkov V.S., Strongin A.Y. (2009). Biochemical evidence of the interactions of membrane type-1 matrix metalloproteinase (MT1-MMP) with adenine nucleotide translocator (ANT): Potential implications linking proteolysis with energy metabolism in cancer cells. Biochem. J..

[136] Loers G., Makhina T., Bork U., Dörner A., Schachner M., Kleene R. (2012). The interaction between cell adhesion molecule L1, matrix metalloproteinase 14, and adenine nucleotide translocator at the plasma membrane regulates L1-mediated neurite outgrowth of murine cerebellar neurons. J. Neurosci..

[137] Kliment C.R., Nguyen J.M.K., Kaltreider M.J., Lu Y., Claypool S.M., Radder J.E., Sciurba F.C., Zhang Y., Gregory A.D., Iglesias P.A. (2021). Adenine nucleotide translocase regulates airway epithelial metabolism, surface hydration and ciliary function. J. Cell Sci..

[138] Marginedas-Freixa I., Alvarez C.L., Moras M., Leal Denis M.F., Hattab C., Halle F., Bihel F., Mouro-Chanteloup I., Lefevre S.D., Le Van Kim C. (2018). Human erythrocytes release ATP by a novel pathway involving VDAC oligomerization independent of pannexin-1. Sci. Rep..

[139] Winkler H.H. (1976). Rickettsial permeability. An ADP-ATP transport system. J. Biol. Chem..

[140] D’Ambrosio A., Zamboni S., Camerini S., Casella M., Sanchez M., Pietraforte D., Vanacore N., Diociauti M., Altieri M., Di Piero V. (2024). Proteomic profile of extracellular vesicles from plasma and CSF of multiple sclerosis patients reveals disease activity-associated EAAT2. J. Neuroinflamm..

[141] Gangoda L., Liem M., Ang C.S., Keerthikumar S., Adda C.G., Parker B.S., Mathivanan S. (2017). Proteomic Profiling of Exosomes Secreted by Breast Cancer Cells with Varying Metastatic Potential. Proteomics.

[142] Lee H.S., Jeong J., Lee K.J. (2009). Characterization of vesicles secreted from insulinoma NIT-1 cells. J. Proteome Res..

[143] Soubannier V., McLelland G.L., Zunino R., Braschi E., Rippstein P., Fon E.A., McBride H.M. (2012). A vesicular transport pathway shuttles cargo from mitochondria to lysosomes. Curr. Biol..

[144] Chamberlain K.A., Huang N., Xie Y., LiCausi F., Li S., Li Y., Sheng Z.H. (2021). Oligodendrocytes enhance axonal energy metabolism by deacetylation of mitochondrial proteins through transcellular delivery of SIRT2. Neuron.

