# Supplementary material for: Adenine Nucleotide Translocase: From Nucleotide Carrier to a Modulator of Mitochondrial Bioenergetics, Quality Control, and Cellular Communication
Source: Cells. 2026 Apr 2;15(7):646. doi: 10.3390/cells15070646 (PMC13072411; doi:10.3390/cells15070646)
Supplement: Supplementary file 1 [file cells-15-00646-s001.zip › cells-4199502-supplementary.pdf]

Suppl. Table S1. Functional Roles of ANT Isoforms Across Cellular Processes

ANT isoform	Tissue expression	Major cellular context	Biological process	Section in the Review
ANT1	Predominantly heart and skeletal muscle; also present in other differentiated tissues	Oxidative, post-mitotic tissues; cardiac stress adaptation	ADP/ATP exchange and oxidative bioenergetics	3.1
ANT1	Heart, skeletal muscle	Oxidative tissues under stress	Mild uncoupling / redox control	3.2
ANT1	Heart, skeletal muscle	Cardiac ischemia/reperfusion and oxidative stress	Cytoprotection / mitochondrial stabilization	3.2; 6.1; 6.5
ANT1	Heart, skeletal muscle; stress-sensitive tissues	Cell death / permeability control	mPTP regulation	4.1
ANT1	Oxidative tissues	Mitochondrial dynamics / network remodeling	Fusion-fission bias	4.2
ANT1	Post-mitotic tissues, especially heart	Mitochondrial quality control	PINK1–Parkin mitophagy	4.3
ANT1	Heart and other post-mitotic tissues	Mitophagy regulation under stress	ANT stabilization / QC	4.3
ANT1	Heart, skeletal muscle	Mitochondrial genome maintenance	mtDNA stability	5.1
ANT1	Differentiated tissues	Nucleoid-associated membrane domains	Genome organization / membrane anchoring	5.2
ANT1	Cardiomyocytes	Intracellular stress signaling	AKT/HSP27/TLR4-linked survival signaling	6.1
ANT1	Cardiomyocytes	miRNA-dependent regulation	Necrotic cell death control	6.2
ANT1	Cardiomyocytes; inflammatory tissues	Cytokine signaling / inflammasome control	NLRP3 restraint / DAMP regulation	6.3
ANT1	Macrophages; ischemic heart	Immune cell metabolism / polarization	M2 polarization / anti-inflammatory adaptation	6.5
ANT1	Cardiomyocytes / secretome context	Tissue-level communication	Paracrine stress adaptation	6.3; 7.2
ANT1	Various cell types (reported ectopically)	Plasma membrane / extracellular space	Extracellular ATP handling	7.1

ANT isoform	Tissue expression	Major cellular context	Biological process	Section in the Review
ANT2	Broadly expressed; enriched in proliferative, glycolytic, and metabolically flexible cells; also muscle	Proliferation / regeneration	ADP/ATP exchange and metabolic flexibility	3.1
ANT2	Proliferative tissues; stressed cardiomyocytes	Hypoxia / impaired OXPHOS	Reverse ATP import / ΔΨm maintenance	3.1
ANT2	Broad distribution including heart and skeletal muscle	Stress adaptation / uncoupling	Mild uncoupling and redox buffering	3.2
ANT2	Heart, stress-adapted tissues	Respiratory adaptation	Respiratory efficiency / abundance effects	3.1
ANT2	Stress-exposed cells	Mitochondrial dynamics / quality control	Fusion competence / fragmentation bias	4.2
ANT2	Post-mitotic and stress-responsive cells	Mitophagy initiation	TIMM23/TIMM44-linked import gating	4.3
ANT2	Injured neurons / axons	Regeneration-associated mitophagy	PINK1 stabilization / mitochondrial clearance	4.3
ANT2	Proliferative tissues	Import stress adaptation	Proteostasis / mitophagy threshold control	4.3
ANT2	Proliferative and regenerative cells	Intracellular signaling / receptor stability	HER2–HSP90 / PI3K–AKT axis	6.1
ANT2	Prostate cancer cells	PTM-dependent survival signaling	PAK6–SIRT4–ANT2 axis	6.1
ANT2	Cancer cells	miRNA-regulated tumor adaptation	Ras/STAT3/PI3K–AKT-linked oncogenic programs	6.2
ANT2	Inflammatory epithelial tissues	Mitochondrial RNA signaling	mtRNA / dsRNA export	6.4
ANT2	Airway epithelium	Inflammatory stress response	ETS2–ANT2–mtRNA axis	6.4
ANT2	T cells	Immune metabolism	Immunometabolic checkpoint control	6.5
ANT2	Myeloid / adipose inflammatory contexts	Metabolic inflammation	Pro-inflammatory macrophage / monocyte signaling	6.5

ANT isoform	Tissue expression	Major cellular context	Biological process	Section in the Review
ANT2	Hepatocytes, tumor cells, airway epithelium	Ectopic localization	Plasma membrane ATP handling / invasion / receptor-linked transport	7.1
ANT2	Developing neurons	Plasma membrane / adhesion signaling	Transient ATP release / neurite outgrowth	7.1
ANT2	Cancer cells, EV datasets	Intercellular communication	EV-associated mitochondrial signaling	7.2
ANT3	Broad ubiquitous expression	Housekeeping / baseline mitochondrial metabolism	Canonical nucleotide exchange	2, 3.1
ANT3	Proliferative and malignant cells	Stress adaptation / proteostasis	PINK1 transport control / mitophagy	4.3
ANT3	Cancer cells	Proteotoxic stress adaptation	Chemoresistance-linked mitophagy	4.3
ANT3	Immune contexts	Cytokine-responsive regulation	IL-4 / IFN-γ-associated signaling	6.3
ANT3	Viral infection	Host–pathogen interaction	Apoptosis suppression	6.2
ANT3	EV proteomics / broad tissue contexts	Intercellular communication	EV-associated mitochondrial protein cargo	7.2
ANT4	Germline / reproductive tissues	Developmentally restricted germ-cell metabolism	ADP/ATP exchange in germ cells	2; 3.1
ANT4	Testis / meiotic cells	Meiotic progression / fertility	Germline energy metabolism	2
ANT4	Reproductive tissues	Epigenetically restricted expression	Developmental specialization	2
